# Supplementary material for: The Glycosphingolipid GM3 Modulates Conformational Dynamics of the Glucagon Receptor
Source: Biophys J. 2020 Jun 17;119(2):300–13. doi: 10.1016/j.bpj.2020.06.009 (PMC7376093; doi:10.1016/j.bpj.2020.06.009)
Supplement: Document S1. Figs. S1–S8 [file mmc1.pdf]

Biophysical Journal, Volume *119*

Supplemental Information

The Glycosphingolipid GM3 Modulates Conformational Dynamics of the Glucagon Receptor

T. Bertie Ansell, Wanling Song, and Mark S.P. Sansom

Supporting Material

Lipid parameters GROMACS *.*itp* files for PIP2 and GM3 are provided as data files.

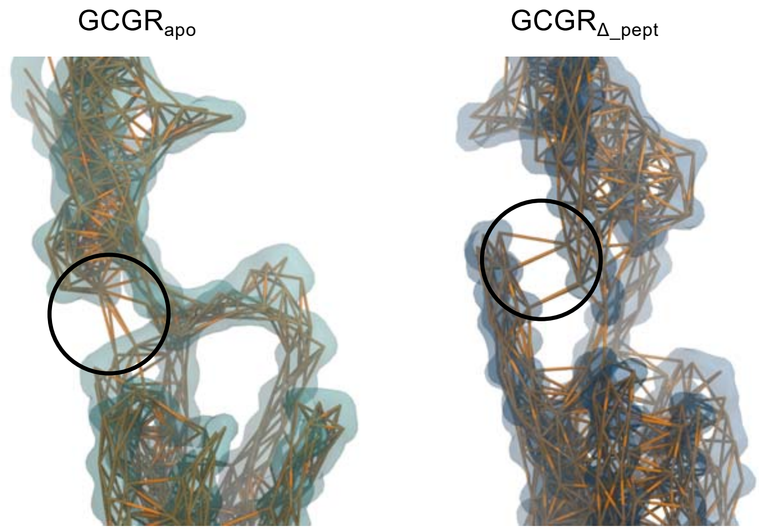

Supplementary Figure S1: Elastic network restraints in CG GCGR structures. Structures of $GCGR_{apo}$ (5XEZ) and $GCGR_{\Delta\_pept}$ (5YQZ) coarse-grained using the MARTINI 2.2 forcefield with an ElNeDyn elastic network (spring force constant = 500 $kJ.mol^{-1}.nm^{-2}$, cut-off = 0.9 nm). The elastic network was visualised using the *cg_bonds.tcl* script with a 0.9 nm cut-off (cgmartini.nl/index.php/tools2/visualization). Circles indicate elastic network restraints connecting the ECD and TMD regions of $GCGR_{apo}$ and $GCGR_{\Delta\_pept}$.

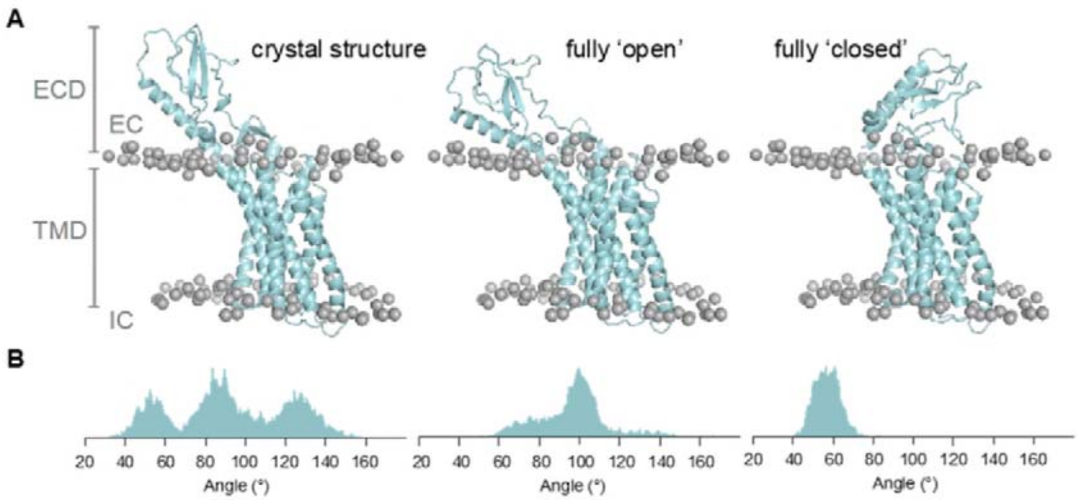

Supplementary Figure S2: Structures used in atomistic simulations. A) Comparison of the initial conformations used in atomistic simulations of $GCGR_{apo}$ (light blue). Each conformation was simulated for 2 x 500 ns in a bilayer composed of POPC (65%): GM3 (10%): CHOL (25%) in the extracellular leaflet and POPC (65%): PIP_2 (10%): CHOL (25%) in the intracellular leaflet. The fully 'open' and fully 'closed' conformations were backmapped from the end of coarse-grain simulations using the *backward.py* and *initram.sh* scripts. Lipid phosphate groups are shown as grey spheres and the position of the extracellular (EC) and intracellular (IC) membranes are marked. **B)** ECD-TMD angle distribution across each of the simulations setups defined as the angle between two planes formed by the Cα beads of R199, V285 and T369 on the TMD, and E34, H45 and H93 on the ECD.

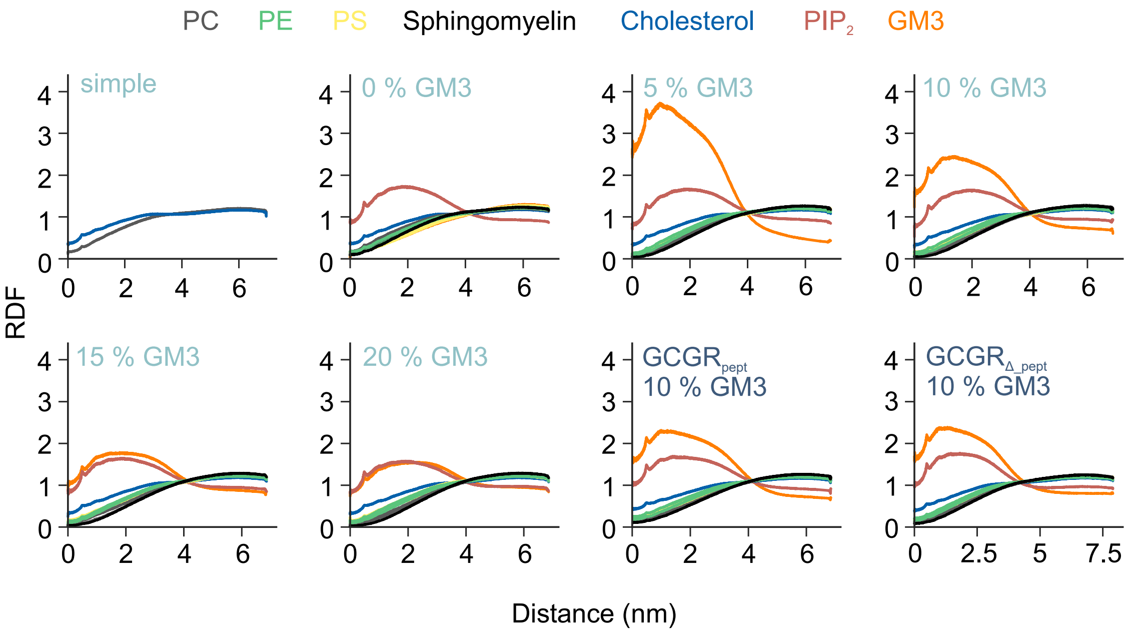

Supplementary Figure S3: Preferential distribution of GM3 and PIP_2 around GCGR. Radial distribution function (RDF) of lipid species surrounding the GCGR TMD centre of geometry in the *xy* plane during CG simulations of $GCGR_{apo}$ (light blue), $GCGR_{pept}$ and $GCGR_{\Delta\_pept}$ (dark blue). $GCGR_{apo}$ was embedded in simple or complex bilayers containing (0-20% GM3). $GCGR_{pept}$ and $GCGR_{\Delta\_pept}$ were embedded in a complex bilayers containing 10% GM3. See Table 1 for a detailed description of lipid compositions and simulation times.

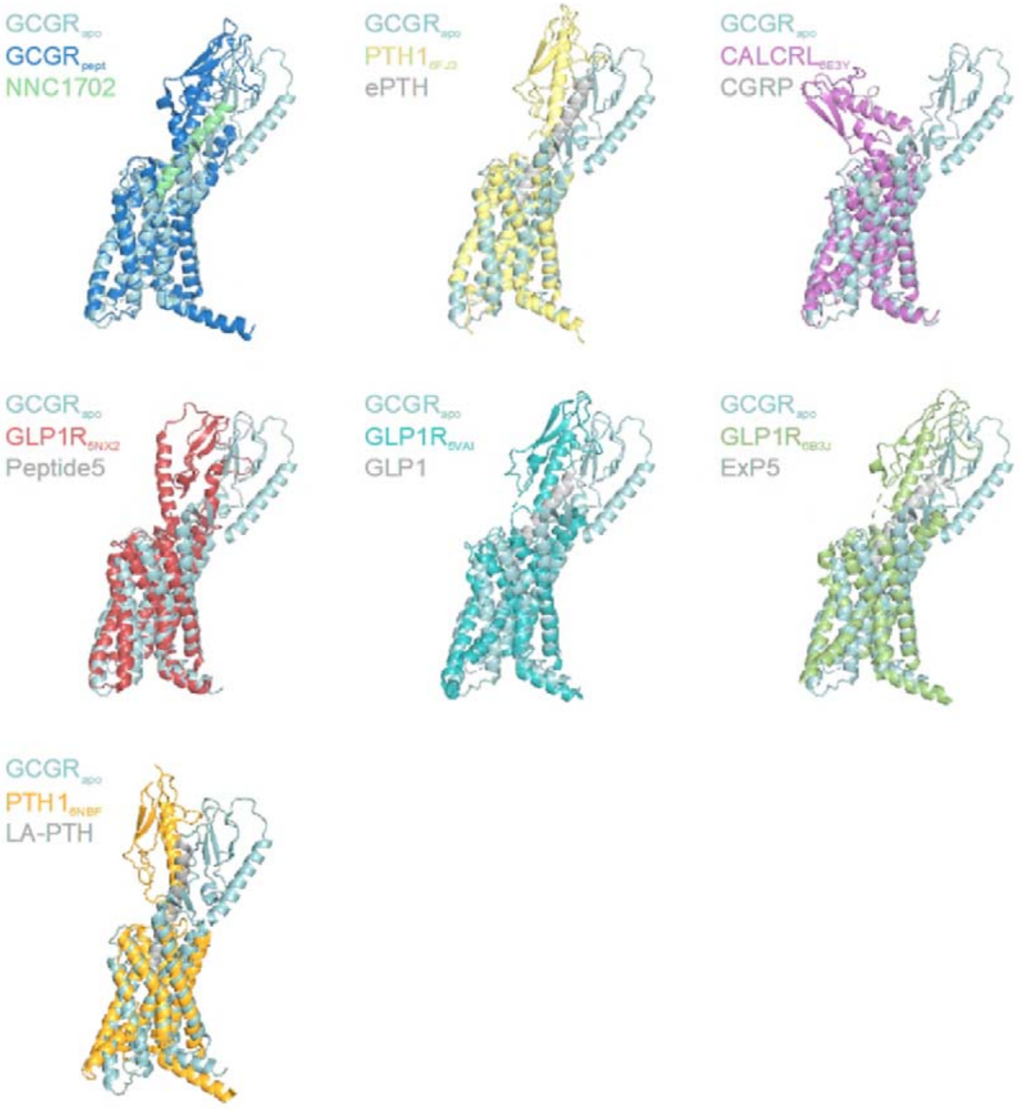

Supplementary Figure S4: Dynamic conformations of Class B1 GPCR ECDs observed in structures. Peptide bound multi-domain Class B1 GPCR structures of the glucagon ($GCGR_{pept}$, PDB: 5YQZ), parathyroid hormone 1 (PTH1, PDBs: 6FJ3, 6NBF), calcitonin receptor-like (CALCRL, PDB: 6E3Y) and glucagon-like receptor 1 (GLP1R, PDBs: 5NX2, 5VA, 6B3J) receptors aligned to the TMD of $GCGR_{apo}$ (PDB: 5XEZ) demonstrating the diversity in ECD position observed experimentally. Peptides bound within the TMD pocket are coloured grey with the exception of NNC1702 which is coloured lime green.

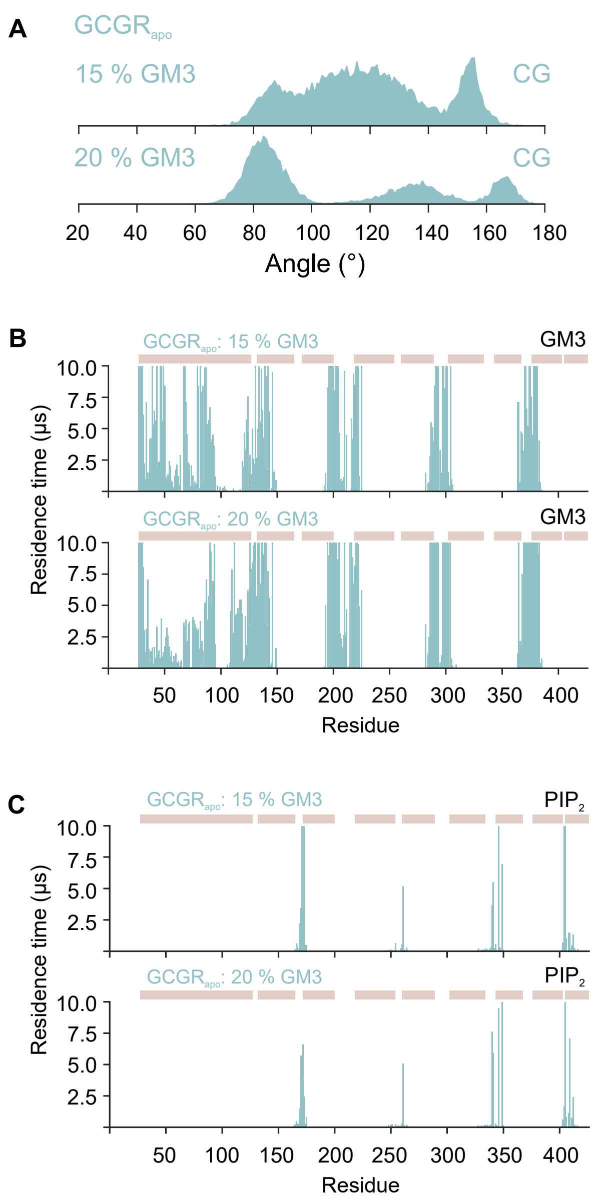

Supplementary Figure S5: ECD dynamics and lipid contacts in bilayers containing 15% or 20% GM3. Analysis derived from CG simulations of $GCGR_{apo}$ embedded in complex bilayers containing 15-20% GM3. **A)** ECD-TMD angle distribution across the simulations defined as the angle between two planes formed by the backbone beads of R199, V285 and T369 on the TMD and E34, H45 and H93 on the ECD. **B)** GM3 and **C)** PIP_2 headgroup interaction profiles with $GCGR_{apo}$ in CG simulations. Lipid headgroup residence times were calculated using an in house procedure (PyLipID) with a 0.55 nm and 1.0 nm dual cut-off scheme. The position of the ECD, TM1-7 and H8 are shown above the contact profile as ochre rectangles.

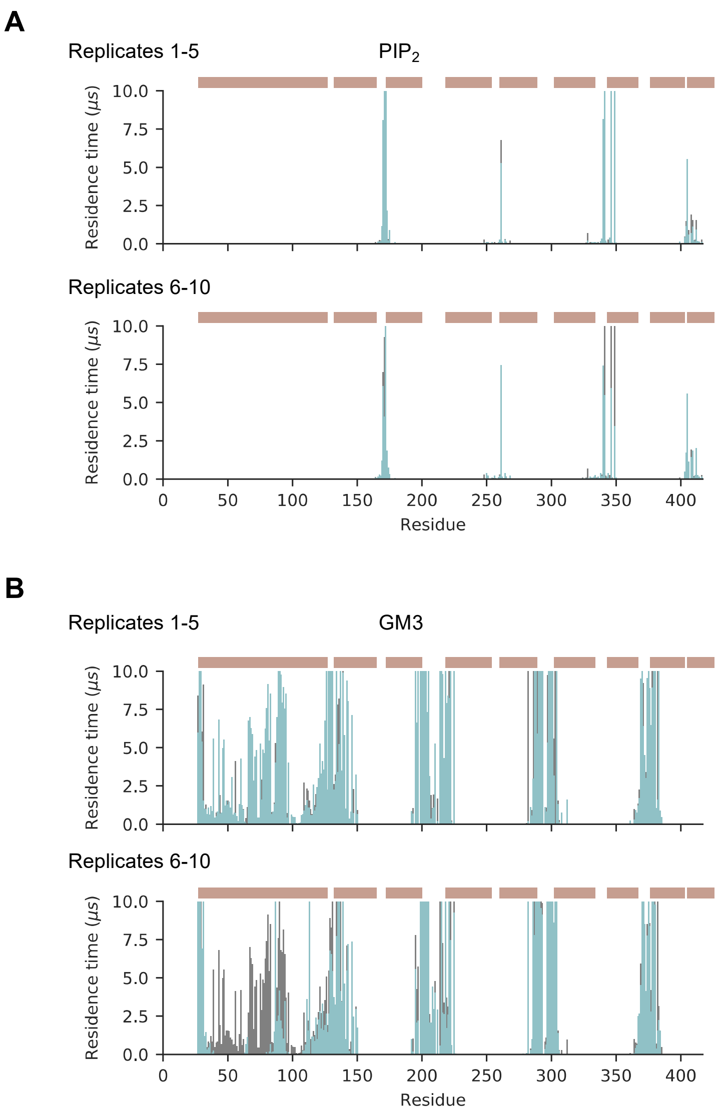

Supplementary Figure S6: Convergence of GM3 and PIP_2 residence times in CG simulations. Lipid interaction residence times of **A)** PIP_2 and **B)** GM3 headgroups from 10 x 10 µs simulations of $GCGR_{apo}$ embedded in a complex bilayer containing 10 % GM3 (grey) overlaid with residence times when simulations were split into two 5 x 10 µs groups (light blue). A 0.55 and 1.0 nm dual interaction cut-off was employed for lipid headgroup contacts analysis. Discrepancies in GM3 headgroup interactions with the ECD in **B** are accounted for by different ECD opening propensities between the two simulation subsets. The position of the ECD, TM1-7 and H8 are shown above the contact profile as ochre rectangles.

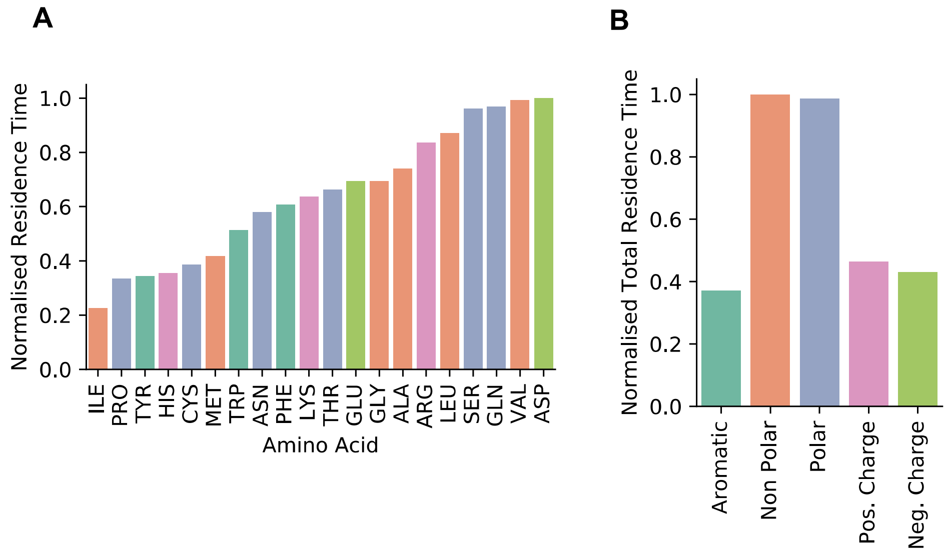

Supplementary Figure S7: Contribution of GCGR amino acid types to GM3 interactions in CG simulations. A) GM3 headgroup residence times for interaction with GCGR amino acids across all CG simulations. Residence times were summed and normalised between 0 and 1 relative to the amino acid with the greatest GM3 headgroup residues time (ASP). Residues are coloured according to physical properties: aromatic (turquoise), non-polar (orange), polar (blue), positively charged (pink) and negatively charged (green). **B)** GM3 residence times grouped according to the physical properties of amino acids. Data for individual amino acids from **A** were grouped according to their physical properties; aromatics (F, Y, W), non-polar (G, A, V, L, M, I), polar (S, T, C, P, N, Q), positively charged (R, K, H) and negatively charged (E, D) and are plotted by total residue type contribution to GM3 interactions. Residence times were normalised between 0 and 1 relative to the amino acid type with the greatest total GM3 headgroup residence times (non-polar).

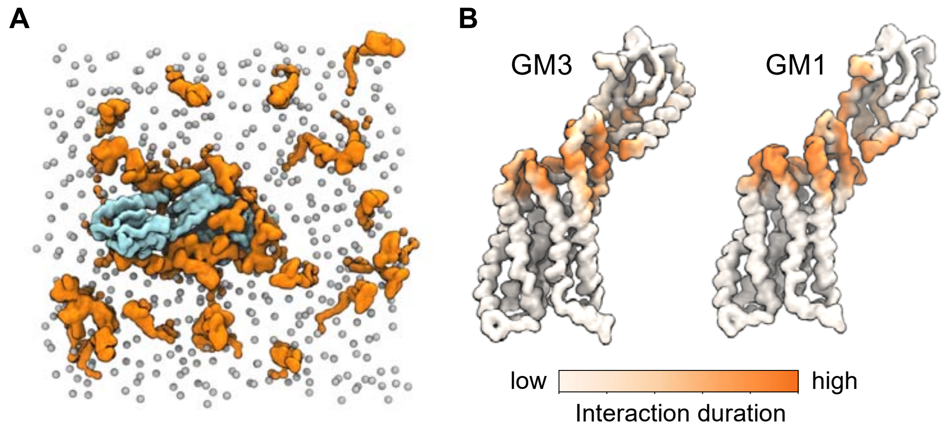

Supplementary Figure S8: Comparison of GM1 and GM3 contacts. CG simulations (3 x 10 μs) of $GCGR_{apo}$ in a 'complex' bilayer were performed whereby 10 % GM1 was used in place of GM3. **A)** Snapshot from the end of one CG simulation showing localisation of GM1 (orange) around $GCGR_{apo}$ (light blue). The membrane patch is shown from above and phosphate groups are shown as grey spheres. **B)** Comparison of GM3 and GM1 contacts mapped onto the structure of $GCGR_{apo}$ showing GM1 interactions over the extracellular TMD regions and the ECD.

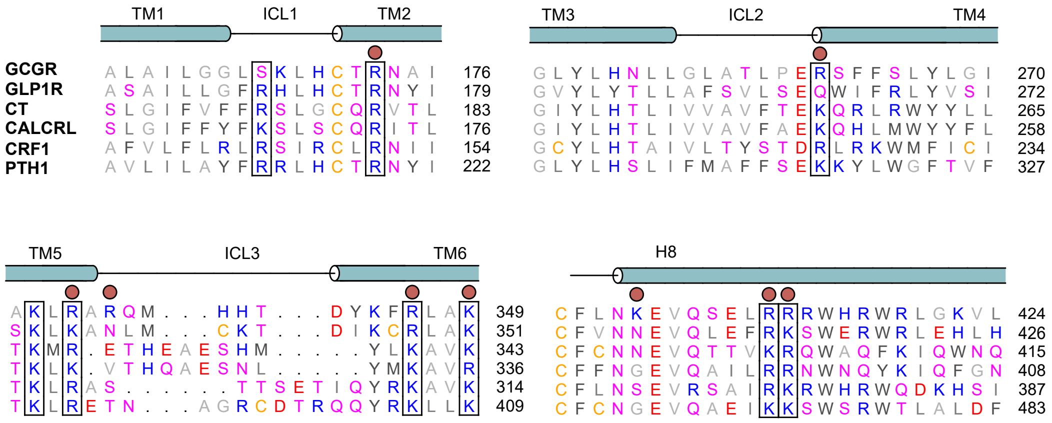

Supplementary Figure S8: Structure-based sequence alignment of the intracellular regions of Class B1 GPCRs. Residues are coloured by residue type and the position of helices in the $GCGR_{apo}$ crystal structure indicated. $GCGR_{apo}$ residues which contribute to PIP_2 binding are marked by red circles. Black rectangles indicate conserved basic residues. Structure based sequence alignment was performed on GPCRdb.org using the human calcitonin (CT), calcitonin receptor-like (CALCRL), corticotropin-releasing factor 1 (CRF1), glucagon-like peptide-1 (GLP1R), glucagon (GCGR) and parathyroid hormone-1 (PTH1) receptors with manual adjustment based on the position of helices observed in structures.
