# Supplementary material for: The Glycosphingolipid GM3 Modulates Conformational Dynamics of the Glucagon Receptor
Source: Biophys J. 2020 Jun 17;119(2):300–13. doi: 10.1016/j.bpj.2020.06.009 (PMC7376093; doi:10.1016/j.bpj.2020.06.009)
Supplement: Document S2. Article plus Supporting Material [file mmc3.pdf]

Biophysical Journal
Article

The Glycosphingolipid GM3 Modulates Conformational Dynamics of the Glucagon Receptor

T. Bertie Ansell,[1] Wanling Song,[1] and Mark S. P. Sansom[1,*]
[1]Department of Biochemistry, University of Oxford, Oxford, United Kingdom

ABSTRACT The extracellular domain (ECD) of class B1 G-protein-coupled receptors (GPCRs) plays a central role in signal transduction and is uniquely positioned to sense both the extracellular and membrane environments. Although recent studies suggest a role for membrane lipids in the modulation of class A and class F GPCR signaling properties, little is known about the effect of lipids on class B1 receptors. In this study, we employed multiscale molecular dynamics simulations to access the dynamics of the glucagon receptor (GCGR) ECD in the presence of native-like membrane bilayers. Simulations showed that the ECD could move about a hinge region formed by residues Q122–E126 to adopt both closed and open conformations relative to the transmembrane domain. ECD movements were modulated by binding of the glycosphingolipid GM3. These large-scale fluctuations in ECD conformation may affect the ligand binding and receptor activation properties. We also identify a unique phosphatidylinositol (4,5)-bisphosphate (PIP_2) interaction profile near intracellular loop (ICL) 2/TM3 at the G-protein-coupling interface, suggesting a mechanism of engaging G-proteins that may have a distinct dependence on PIP_2 compared with class A GPCRs. Given the structural conservation of class B1 GPCRs, the modulatory effects of GM3 and PIP_2 on GCGR may be conserved across these receptors, offering new insights into potential therapeutic targeting.

SIGNIFICANCE The role of lipids in regulation of class B G-protein-coupled receptors (GPCRs) remains elusive, despite recent structural advances. In this study, multiscale molecular dynamics simulations are used to evaluate lipid interactions with the glucagon receptor, a class B1 GPCR. We find that the glycosphingolipid GM3 binds to the glucagon receptor extracellular domain, modulating the dynamics of the extracellular domain and promoting movement away from the transmembrane domain. We also identify a unique phosphatidylinositol (4,5)-bisphosphate interaction fingerprint in a region known to be important for bridging G-protein coupling in class A GPCRs. Thus, this study provides molecular insight into the behavior of the glucagon receptor in a mixed lipid bilayer environment, which may aid understanding of glucagon receptor signaling properties.

INTRODUCTION

Class B1 G-protein-coupled receptors (GPCRs) are involved in a diverse range of signaling pathways, including calcium homeostasis, metabolism, and angiogenesis (1). Class B1 receptors are composed of a canonical GPCR seven transmembrane helix bundle (TM1–7), a C-terminal membrane-associated helix (H8), and an N-terminal 120–160 residue extracellular domain (ECD). The ECD has a conserved fold (2) and plays a key role in peptide ligand binding, signal transduction, and signaling specificity (3). A "two-domain" binding mechanism for peptide ligands has been proposed for class B1 GPCRs whereby rapid binding of the C-terminus of the peptide to the ECD precedes slower insertion of peptide N-terminus into the transmembrane domain (TMD), leading to conformational rearrangements and receptor activation (4). Differences in the requirement of the ECD for receptor signaling and ligand binding may exist across the class B1 family. For the polypeptide-type 1 (PAC1R), parathyroid hormone (PTH1R), and corticotrophin-releasing factor 1 (CRF1R) receptors, the requirement for the ECD can be bypassed by mass action effects or hormone tethering, consistent with the "two-domain" model and the role of the ECD as an affinity trap. In contrast, for the glucagon receptor (GCGR) and glucagon-like peptide-1 receptor (GLP1R), the ECD is required for receptor signaling even when the ligand is tethered to the TMD, complicating interpretation of the "two-domain" model (5).

The GCGR is a class B1 GPCR involved in regulation of glucose homeostasis and amino acid and lipid metabolism

Submitted March 13, 2020, and accepted for publication June 9, 2020.
*Correspondence: mark.sansom@bioch.ox.ac.uk
Editor: Ilya Levental.
https://doi.org/10.1016/j.bpj.2020.06.009
© 2020 Biophysical Society.
This is an open access article under the CC BY license (http://creativecommons.org/licenses/by/4.0/).

(6–8). Consequently, the GCGR is a potential candidate for treatment of diseases associated with insulin resistance, such as metabolic syndrome or type 2 diabetes, the prevalence of which increased twofold over the past 30 years (9). Structures of the full-length GCGR have revealed distinct conformations of the ECD, which differ by rotation around a hinge region linking the ECD to the TMD (10,11). Hydrogen-deuterium exchange experiments alongside MD simulations suggest the GCGR ECD is mobile and can form TMD contacts in the absence of bound ligand (10,12), further implicating ECD plasticity as a key attribute in GCGR function. Furthermore, a combination of cryoelectron microscopy and MD simulations suggest ECD mobility may be required for binding of peptide ligand to the related GLP1R (13). However, the role of lipids in activation of class B1 GPCRs is less well understood. While the activation of class A GPCRs is modulated by membrane lipids (14–16) which may act as allosteric regulators of GPCR activity (15,17,18), the interactions of lipids with class B GPCRs have not been extensively characterized.

Molecular dynamics (MD) simulations enable exploration of how the physical properties of a membrane (19) and/or direct lipid interactions (20–22) may alter the conformational dynamics of membrane proteins (15,23). For example, a crystal structure of the class A GPCR β_2-adrenergic receptor identified cholesterol bound to the intracellular region of TM4 (24), which was validated by observation of cholesterol binding to the same binding site in MD simulations (25,26). MD simulations have shown how cholesterol binding can modulate the conformation dynamics of the β2-adrenergic receptor (27). MD simulations have also demonstrated that phosphatidylinositol (4,5)-bisphosphate (PIP_2) binds more favorably to active than to inactive states of the adenosine 2A receptor, thus favoring receptor activation (28).

Simulations of GCGR have thus far been limited to bilayers containing just the neutral lipid phosphatidylcholine (PC) (10,12,29,30). Given the role of lipids in GPCR regulation, it is therefore timely to explore the interactions of GCGR with mixtures of lipids, mimicking cellular membranes (31,32). Furthermore, given the proximity of the ECD to the outer leaflet of the plasma membrane, we wished to establish whether an asymmetric and relatively complex lipid environment could influence the dynamics of the ECD relative to the TMD. In particular, given that gangliosides such as GM3 interact with and allosterically regulate other classes of receptor (e.g., the EGF receptor (33,34)), we wished to explore whether GM3 could interact with the GCGR. Using a multiscale MD simulation approach, we combine exploration of protein-lipid interactions via coarse-grained (CG) MD simulations with the more detailed representation of interactions in atomistic simulations to probe GCGR dynamics and lipid interactions in in vivo mimetic membrane environments.

METHODS

Coarse-grained MD simulations

Simulations were performed using GROMACS 5.1.4 (www.gromacs.org). GCGR structures were derived from Protein Data Bank identifiers PDB: 5XEZ and PDB: 5YQZ (10,11). The T4-lysozyme insert was removed from intracellular loop 2 (ICL2), and residues between A256 and E260 (5XEZ) or T257 and E260 (5YQZ) were modeled using MODELER 9.19 (35). The *martinizy.py* script was used to make the receptor coarse grained (36). For the 5YQZ structure, the receptor and peptide were coarse grained separately before consolidation. The ElNeDyn elastic network with a spring force constant of 500 kJ $\cdot$ mol^{-1} $\cdot$ nm^{-2} and cutoff of 0.9 nm was applied (37). Minimal elastic network restraints were present between the ECD and TMD because of the hinge-like arrangement of these domains in the 5XEZ and 5YQZ structures (Fig. S1). The transmembrane region of GCGR was embedded in the bilayer using *insane.py* (38), and the receptor centered in a 15 $\times$ 15 $\times$ 17 nm^3 box. Details of the mixtures of lipids employed are given in Table 1. For the lipids, we used the most recent **.itp* files compatible with MARTINI 2.2, along with the virtual sites cholesterol model (39). The system was solvated using MARTINI water (40) and 150 mM NaCl. Each replicate was independently set up before being subjected to energy minimization using the steepest-descent method. Each system was then equilibrated in the NPT ensemble for two 100-ns runs with restraints applied to all protein beads during the first step and just to backbone beads during the second step.

CG simulations were run for 10 μs with a 20-fs integration time step using the MARTINI 2.2 force field to describe all components (36,41). Five or 10 repeat simulations of the GCGR structures (5XEZ or 5YQZ) in combination with each of the bilayer compositions as specified in Table 1 were performed, totaling 700 μs of CG simulation data. Temperature was maintained at 323 K using the V-rescale thermostat (42) and a coupling constant $\tau_t = 1.0$ ps. Pressure was maintained at 1 bar using the Parrinello-Rahman barostat (43), a coupling constant $\tau_p = 12.0$ ps, and a compressibility of 3 $\times$ 10^{-4} bar^{-1}. The reaction field method was used for Coulomb interactions with a cutoff value of 1.1 nm. van der Waals interactions were cut off at 1.1 nm using the potential-shift Verlet method. The LINCS algorithm (44) was used to constrain bonds to their equilibration values.

Protein-lipid interactions were analyzed using an in-house procedure (PyLipID; https://github.com/wlsong/PyLipID) to calculate the residence time of lipid interactions with GCGR in CG simulations. Briefly, lipid contacts were initiated when any of lipid headgroup beads came within 0.55 nm of the CG protein surface and ended when they exceeded 1.0 nm. In atomistic simulations, a dual cutoff of 0.35–0.55 nm was used. Bi-exponential curve fitting of lipid interaction durations as a function of time were used to estimate k_{off} values for lipid interactions. These k_{off} values were used to derive lipid residence times, which form the basis of the interaction profiles shown in the figures.

Atomistic MD simulations

For atomistic simulations, the protein structure was embedded in lipid bilayers, which were assembled using the CHARMM-GUI bilayer builder (45). Atomistic bilayers were composed of POPC (65%): GM3 (10%) and cholesterol (25%) in the extracellular leaflet and POPC (65%): PIP_2 (10%) and cholesterol (25%) in the intracellular leaflet. The default atomic charges for GM3 (overall charge = -1) and PIP_2 (overall charge = -4) as specified in the CHARMM-GUI output were used, the parameterization of which is described elsewhere, yielding experimentally comparable bilayer systems (46,47). GM3 and PIP_2 itp files are provided as Data S1. The GROMACS 4.6 *g_membed* tool (48) was used to embed GCGR in a bilayer before solvation using TIP3P water (49) and 150 mM NaCl. For each replicate, independent steepest-descent energy minimization followed by 5-ns NVT and NPT equilibration steps were performed with restraints applied to the protein.

TABLE 1 Summary of Simulations

Protein	CG or AT	Name	Lipid Composition[a]	Replicates × Duration
$GCGR_{apo}$	CG	Binary mixture	POPC, Chol 3:1	10 × 10 μs
$GCGR_{apo}$	CG	Mixed lipid: 0% GM3	EC – POPC, DOPC, POPE, DOPE, Sph[b], CHOL 25:25:5:5:15:25 IC – POPC, DOPC, POPE, DOPE, POPS, DOPS, PIP_2, CHOL 5:5:20:20:8:7:10:25	10 × 10 μs
$GCGR_{apo}$	CG	Mixed lipid: 5% GM3	EC – POPC, DOPC, POPE, DOPE, Sph[b], GM3[b], CHOL 22.5:22.5:5:5:15:5:25 IC – POPC, DOPC, POPE, DOPE, POPS, DOPS, PIP_2, CHOL 5:5:20:20:8:7:10:25	10 × 10 μs
$GCGR_{apo}$	CG	Mixed lipid: 10% GM3	EC – POPC, DOPC, POPE, DOPE, Sph[b], GM3[b], CHOL 20:20:5:5:15:10:25 IC – POPC, DOPC, POPE, DOPE, POPS, DOPS, PIP_2, CHOL 5:5:20:20:8:7:10:25	10 × 10 μs
$GCGR_{apo}$	CG	Mixed lipid: 10% GM1	EC – POPC, DOPC, POPE, DOPE, Sph[b], GM1[b], CHOL 20:20:5:5:15:10:25 IC – POPC, DOPC, POPE, DOPE, POPS, DOPS, PIP_2, CHOL 5:5:20:20:8:7:10:25	3 × 10 μs
$GCGR_{apo}$	CG	Mixed lipid: 15% GM3	EC – POPC, DOPC, POPE, DOPE, Sph[b], GM3[b], CHOL 17.5:17.5:5:5:15:15:25 IC – POPC, DOPC, POPE, DOPE, POPS, DOPS, PIP_2, CHOL 5:5:20:20:8:7:10:25	5 × 10 μs
$GCGR_{apo}$	CG	Mixed lipid: 20% GM3	EC – POPC, DOPC, POPE, DOPE, Sph[b], GM3[b], CHOL 15:15:5:5:15:20:25 IC – POPC, DOPC, POPE, DOPE, POPS, DOPS, PIP_2, CHOL 5:5:20:20:8:7:10:25	5 × 10 μs
$GCGR_{pept}$	CG	Mixed lipid: 10% GM3	EC – POPC, DOPC, POPE, DOPE, Sph[b], GM3[b], CHOL 20:20:5:5:15:10:25 IC – POPC, DOPC, POPE, DOPE, POPS, DOPS, PIP_2, CHOL 5:5:20:20:8:7:10:25	10 × 10 μs

(*Continued in next column*)

Table 1. *Continued*

Protein	CG or AT	Name	Lipid Composition[a]	Replicates × Duration
$GCGR_{\Delta\_pept}$	CG	Mixed lipid: 10% GM3	EC – POPC, DOPC, POPE, DOPE, Sph[b], GM3[b], CHOL 20:20:5:5:15:10:25 IC – POPC, DOPC, POPE, DOPE, POPS, DOPS, PIP_2, CHOL 5:5:20:20:8:7:10:25	10 × 10 μs
$GCGR_{apo}$	AT	Mixed lipid: 10% GM3	EC – POPC, (18:1/16:0) GM3, CHOL 65:10:25 IC – POPC, PIP_2, CHOL 65:10:25	2 × 0.5 μs

EC, extracellular leaflet; IC, intracellular; Sph, sphingomyelin.
[a]PIP_2 was modeled with 1-palmitoyl-2-oleoyl tails.
[b]Lipids with C(d18:1/18:0) N-stearoyl-D-erythro tails.

Two 500-ns atomistic simulations were run for each initial protein conformation, totaling 3 μs of atomistic data (Table 1; also see Fig. S2). GROMACS 5.1.4 (www.gromacs.org) was used to perform atomistic simulations. A 2-fs time step was used, and the CHARMM-36 force field was used to describe all components (50). Long-range electrostatics were modeled using the particle mesh Ewald model (51), and van der Waals interactions were modeled with cutoff type and rvdw = 1.2 nm. A dispersion correction was not applied. Temperature was maintained at 323 K using the Nosé-Hoover thermostat (52,53) with a coupling constant $\tau_t = 0.5$ ps. Pressure was maintained at 1 bar using the Parrinello-Rahman barostat (43), a coupling constant $\tau_p = 2.0$ ps, and a compressibility of 4.5×10^{-5} bar^{-1}. All bonds were constrained using the LINCS algorithm (44).

All analyses were carried out using GROMACS 5.1 tools (www.gromacs.org) and locally developed scripts. VMD (54) and PyMOL (55) were used for visualization.

RESULTS AND DISCUSSION

GM3 and PIP_2 are preferentially localized around GCGR

We wished to explore the effect of lipid bilayer composition on ECD dynamics, given the dynamic behavior of GCGR observed in previous atomistic simulations (10,12,30) and the proximity of the ECD to the extracellular leaflet of the bilayer. We therefore performed CG MD simulations of the apo-state of GCGR ($GCGR_{apo}$, corresponding to PDB:5XEZ; see Fig. 1 *A* and Methods), of the receptor with a bound glucagon analog and partial agonist peptide NNC1702 ($GCGR_{pept}$, corresponding to 5YQZ) and of the latter state with the NNC1702 peptide removed ($GCGR_{\Delta\_pept}$). All three structures were simulated in a mixed and asymmetric lipid bilayer (PC (40%): PE (10%): sphingomyelin (15%): GM3 (10%): cholesterol (25%) in the extracellular leaflet; PC (10%): PE (40%): PS (15%): PIP_2 (10%): cholesterol (25%) in the intracellular leaflet), chosen to approximate the composition of the plasma

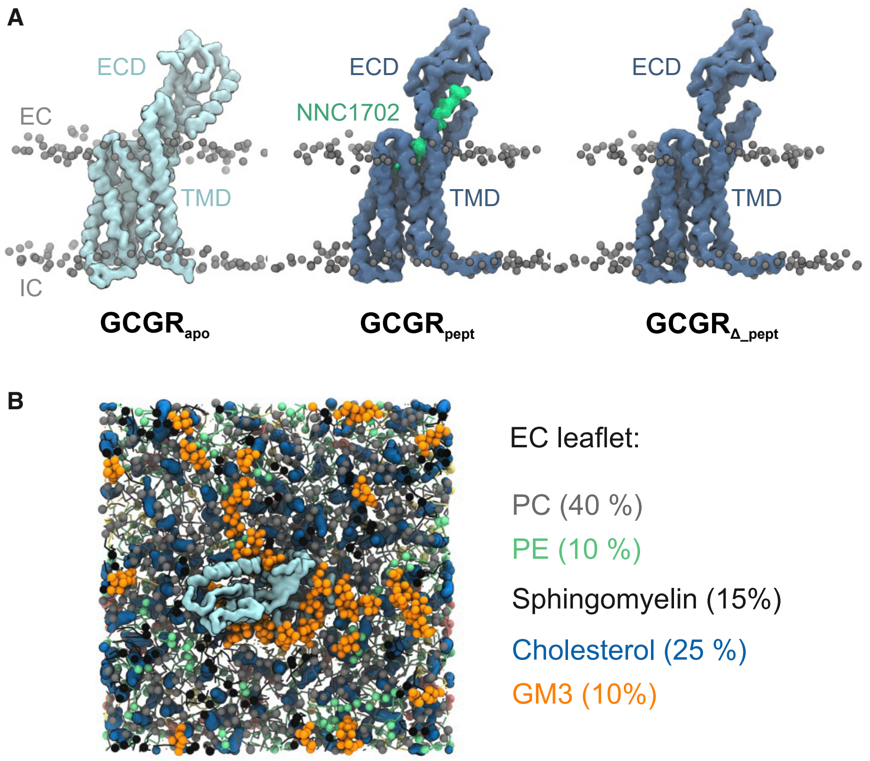

FIGURE 1 Glucagon receptor (GCGR) structures and lipid bilayer composition. (*A*) CG representation of three different GCGR conformations corresponding to GCGR$_{apo}$ (PDB: 5XEZ), GCGR$_{pept}$ (PDB: 5YQZ), and GCGR$_{\Delta_pept}$ (PDB: 5YQZ, devoid of peptide agonist NNC1702). GCGR backbone beads are shown as quicksurf representations and are colored light blue (GCGR$_{apo}$) and dark blue (GCGR$_{pept}$/GCGR$_{\Delta_pept}$). Lipid phosphate groups of the extracellular (EC) and intracellular (IC) leaflets are shown as gray spheres, and the NNC1702 peptide is colored lime green. (*B*) CG representation of a GCGR$_{apo}$ molecule embedded in a 15 × 15 nm^2 "complex" asymmetric bilayer viewed from the extracellular leaflet. Lipids colors are as follows: PC (*gray*), PE (*mint*), sphingomyelin (*black*), cholesterol (*blue*), and GM3 (*orange*). To see this figure in color, go online.

membrane (Fig. 1 *B*). This mixed lipid model is a compromise between computational simplicity (31) and the complexities of modeling realistic cell membrane environments based on lipidomics and related data (32,56). For each simulation condition (see Table 1), 10 replicates each of 10-μs duration were performed. Previous studies of GPCRs have suggested this is sufficient to adequately sample protein-lipid interactions (14).

Comparison of the radial distribution of lipid species surrounding the receptor TMD showed preferential localization of GM3 and PIP_2 in the mixed lipid bilayers (Fig. S3) compared with other lipid species. A locally high radial distribution of GM3 and PIP_2 has been observed previously for simulations of class A receptors (28,57), and PIP_2 binding has been seen during simulations of the class F GPCR Smoothened (58). Bound PIP_2 molecules have also been seen in a recent cryo-EM structure of neurotensin receptor 1 (16). However, the current study is the first observation of increased localization of GM3 and PIP_2 surrounding a class B1 GPCR to the best of our knowledge.

Open and closed conformations of the ECD

Because the ECD of class B1 GPCRs plays a key role in peptide capture and receptor signal transduction (3), we sought to characterize GCGR ECD conformational behavior in native-like membranes. This was aided by the two crystal structures of GCGR (5XEZ and 5YQZ) having ECD conformations that differ by an ~90° rotation (10,11). The ECD of GCGR$_{apo}$ has a distinct conformation compared with that of GCGR$_{pept}$, which represents the canonical peptide-bound conformation as seen in several class B1 GPCRs (Fig. S4; (59–66)). The stalk in GCGR$_{apo}$ also forms a β-sheet with extracellular loop 1 (ECL1). This may be unique to the GCGR apo-state or may be a consequence of the inhibitory antibody fragment (mAb1) used in crystallization, which binds the ECD and ECL1 (10).

In our CG MD simulations of GCGR in mixed lipid membranes (Fig. 2), we observed movement of GCGR$_{apo}$ ECD away from the TMD toward the bilayer (which we will refer to as ECD "opening"), around a hinge region formed by residues Q122-E126. This motion permits ECD contact with the bilayer. We also observed movement of the ECD toward the TMD (ECD "closure"), consistent with observations in published atomistic simulations in a simple PC bilayer (10).

Given the increase in bilayer complexity compared with previous simulations, and our observation (above) of GM3 localization around GCGR, we postulated that ECD opening and/or closing may occur as a result of changes in contacts with the headgroup of the ganglioside (Fig. 2). To investigate the potential influence of GM3 on GCGR$_{apo}$ ECD behavior, we performed CG simulations in both a binary mixture bilayer composed of POPC/CHOL (3:1) (Fig. 2 *C*; Table 1) and in more complex mixed lipid bilayers in which the abundance of GM3 was modulated between 0 and 10%, adjusting the amount of PC accordingly (Table

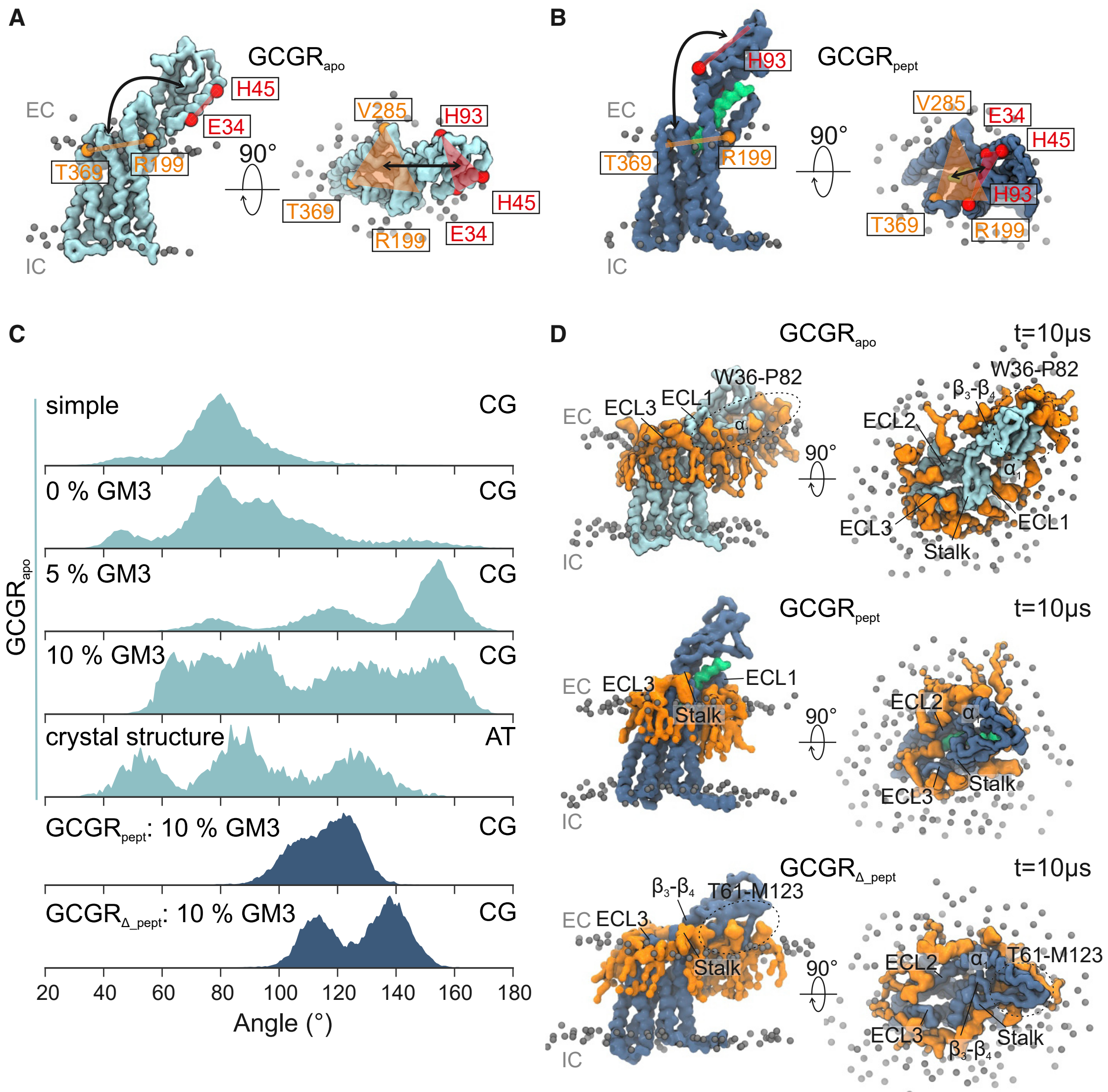

FIGURE 2 GM3 promotes opening of GCGR ECD toward the bilayer. (*A* and *B*) The angle between two planes (defined by the backbone beads of R199, V285, and T369 on the TMD in *orange*, and E34, H45, and H93 on the ECD in *red*) characterizes the conformation of the ECD relative to the TMD. These ECD-TMD planes are indicated on CG structures of (*A*) $GCGR_{apo}$ (*light blue*) and (*B*) $GCGR_{pept}$ (*dark blue*). The NNC1702 peptide bound to $GCGR_{pept}$ is colored lime green. Lipid phosphate groups are shown as gray spheres, and the position of the extracellular (EC) and intracellular (IC) leaflets are indicated. (*C*) ECD-TMD angle distribution is calculated across simulations. Simulations from the top down correspond to CG simulations of $GCGR_{apo}$ embedded in a binary mixture (labeled "simple") POPC/cholesterol bilayer or complex bilayers (as shown in Fig. 1 B), containing 0, 5, or 10% GM3, to atomistic simulations of $GCGR_{apo}$ embedded in mixed lipid bilayers containing 10% GM3 with the initial protein conformation set to the crystal structure, and to CG simulations of $GCGR_{pept}$ and $GCGR_{\Delta\_pept}$ in mixed lipid bilayers containing 10% GM3. (*D*) GM3 (*orange*) bound to $GCGR_{apo}$, $GCGR_{pept}$, and $GCGR_{\Delta\_pept}$ at the end of CG simulations in mixed lipid bilayers containing 10% GM3. Extracellular loops and regions of the ECD interacting with GM3 are labeled. To see this figure in color, go online.

1). We also performed simulations in mixed lipid bilayers containing an enhanced content of GM3 (15 and 20%, compared with the physiological plasma membrane GM3 concentration of ~10% (67)) to mimic possible lateral fluctuations in the local GM3 content of cell membranes (Fig. S5 A; (68)).

To describe motions of the ECD in simulations of the GCGR with different bilayer compositions, we calculated the angle between two planes defined by the residues E34, H45, and H93 on the ECD and R199, V285, and T369 on the TMD (Fig. 2, *A* and *B*). For $GCGR_{apo}$ in either a binary mixture bilayer or a more complex mixed lipid bilayer lacking GM3, the mean (± standard deviation) angles between the ECD and TMD planes were 79° (±16°) and 90° (±25°), respectively. Inclusion of GM3 in the mixed lipid bilayer increased the mean angle to 135° (±27°) and 109° (±32°) for 5 and 10% GM3, respectively (Fig. 2 *C*). A shift in the distribution of ECD-TMD angles to angles >120° when GM3 is included in the bilayer is consistent with the ability for GM3 to promote a greater range of

$GCGR_{apo}$ ECD movement. The increased variability of the ECD-TMD angles when GM3 is included is reflected by a higher standard deviation compared with in the absence of GM3.

Compared with $GCGR_{apo}$, ECD motions were drastically reduced for $GCGR_{pept}$, resulting from peptide contacts bridging the TMD and ECD, which restricted domain movement around the hinge region. In the absence of the peptide (simulation $GCGR_{\Delta\_pept}$), the ECD was observed to move toward the membrane in a manner distinct from that in $GCGR_{apo}$. Visualization of the trajectories revealed that the ECD closing conformation was maintained by interactions of GM3 with ECD loop W106-A118 and the opening conformation by interactions of GM3 with regions focusing around the α_1-helix (Fig. 2 *D*). We calculated the ECD-TMD angle for $GCGR_{pept}$ and $GCGR_{\Delta\_pept}$ and, although not directly comparable to $GCGR_{apo}$ because of the 90° ECD rotation in the crystal structures, we observed an increase in the mean ECD-TMD angle from 116° (±10°) for $GCGR_{pept}$ to 128° (±14°) for $GCGR_{\Delta\_pept}$ (Fig. 2 *C*). This suggests that for both crystal structures, the ECD conformations are inherently flexible (when devoid of bound peptide) and share a propensity to move toward the membrane. Comparison of the distributions of GM3 around $GCGR_{apo}$, $GCGR_{pept}$, and $GCGR_{\Delta\_pept}$ at the end of CG simulations in mixed lipid bilayers containing 10% GM3 revealed GM3 binding to the ECD of $GCGR_{apo}$ and $GCGR_{\Delta\_pept}$ but not to $GCGR_{pept}$ (Fig. 2 *D*). This suggests that changes in the conformation of the receptor may be linked to GM3 binding in the absence of bound peptide. Given the structural conservation of the ECD, ganglioside-mediated modulation of ECD dynamics might be expected to occur in other class B1 GPCRs. This in turn could modulate interactions with peptide ligands and/or bias the receptor conformation toward a particular state via sensing of the local bilayer composition.

ECD movements in atomistic simulations

We have observed ECD movements in multi-microsecond CG simulations, even though an elastic network is present in such simulations (37). To investigate the robustness of these results to the granularity of the simulations, we also performed atomistic simulations (2 × 0.5 μs; Table 1) of $GCGR_{apo}$ starting from the conformation present in the crystal structure. The mean ECD-TMD angle was 92° (±29°), i.e., the ECD behavior in this case showed a mean angle similar to that in CG simulations in binary mixture or more complex mixed lipid bilayers with a low GM3 content. The extent of lipid diffusion during the atomistic simulations allows for just a limited number of GM3 contacts to (re)form with the ECD. Despite this, a peak was observed for ECD-TMD angles >120° (Fig. 2 *C*), and the standard deviation of ECD-TMD angles was high, suggesting GM3 has a similar effect on the ECD-TMD angle distribution at atomistic resolution.

In one of the atomistic simulations initiated from the $GCGR_{apo}$ crystal structure, we observed closure and subsequent reopening of the ECD as the simulation progressed (Fig. 3). We analyzed the ECD motions along with GM3 headgroup binding to two regions on the ECD (site 1 and site 2) over the course of both atomistic simulations (Fig. 3, *A–C*). In one simulation, GM3 molecules were initially bound to site 1 on the $GCGR_{apo}$ ECD, and the ECD-TMD angle fluctuated around ~130°. Loss of these GM3 contacts resulted in closure of the ECD toward the TMD (seen as a decrease in angle and increase in RMSD) from 80 to 410 ns. GM3 subsequently rebound to site 1, resulting in reopening and an increase in the ECD-TMD to 130°. In the second simulation, site 1 was initially occupied by GM3. Again, dissociation of GM3 from site 1, and subsequent binding of GM3 at site 2, was accompanied by closure of the ECD. Binding of GM3 at site 2 locked the ECD in a closed conformation and prevented reopening of the ECD over the course of the simulation. Taken together, these results suggest that interactions of GM3 promote receptor opening, but that this may be modulated by contacts at site 2 which in turn may favor closure. Furthermore, these observations from the atomistic simulations suggest ECD opening/closure is accessible on the sub-microsecond timescale and that stable contacts to GM3 at site 1 are able to maintain an open conformation of the GCGR.

To further compare the conformational dynamics of GCGR in both atomistic and CG simulations in mixed lipid bilayers containing 10% GM3, we performed principal component analysis using trajectories fitted to the TMD (Fig. 4). For $GCGR_{apo}$, the motions of the ECD accounted for by the first principal component were comparable in the CG and atomistic simulations, corresponding to opening and closure of the ECD around the hinge region. The first principal component ranged from 21 to 85% of the total motion (from the component eigenvalues) across the CG simulation replicates and 23–84% in atomistic simulations. In contrast, for $GCGR_{\Delta\_pept}$, movement accounted for by the first principal component shows ECD tilting such that the W106-A118 loop approaches the bilayer, accounting for 24–63% of total component eigenvalues. Although movement represented by the first principal component of $GCGR_{pept}$ ECD was generally characterized by W106-A118 loop movement toward the bilayer, comparable to $GCGR_{\Delta\_pept}$, there were small differences in the extent and angle of ECD movement between replicates, suggesting that the presence of bound peptide alters the propensity of the ECD to move toward the bilayer. These eigenvalues accounted for 19–61% for $GCGR_{pept}$, slightly lower than those of $GCGR_{apo}$ and $GCGR_{\Delta\_pept}$.

Taken together, our results indicate that interactions of GM3 with different regions of the ECD may lead to diverse ECD conformational dynamics. The interactions of GM3

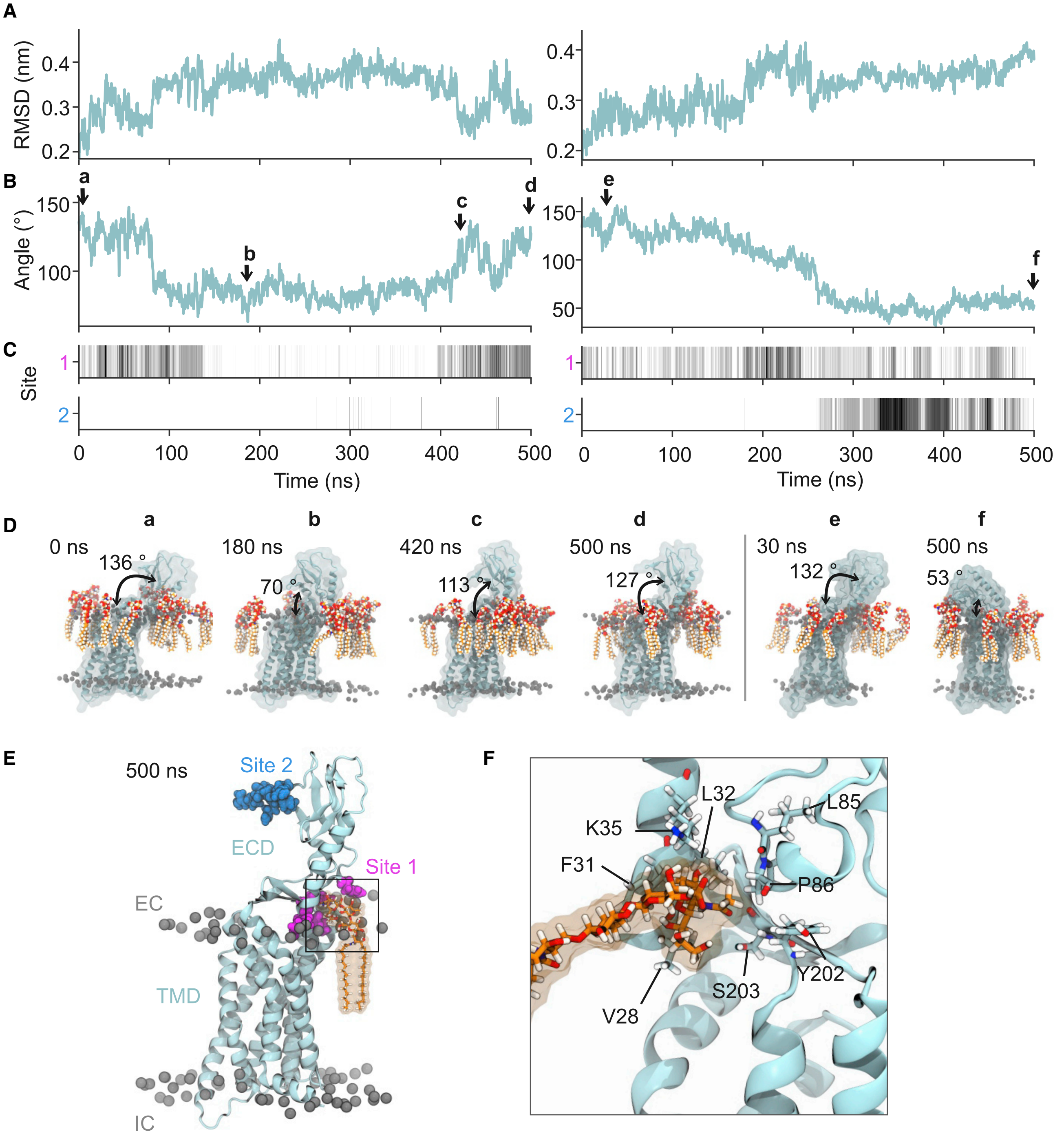

FIGURE 3 GM3 binding modulates movement of the $GCGR_{apo}$ ECD. (*A*) The all-atom RMSD of the ECD (residues 27–132) across simulations superimposed on the TMD and (*B*) the ECD-TMD angle changes as a function of time for two 500-ns atomistic simulations of $GCGR_{apo}$ initiated from the crystal structure conformation in the presence of 10% GM3 (see Table 1). In (*B*) the ECD-TMD angle was defined as the angle between two planes formed by the Cα atoms of R199, V285, and T369 on the TMD and E34, H45, and H93 on the ECD. Arrows indicate snapshots at (a to d) t = 0, 180, 420, and 500 ns for the first repeat simulation and at (e and f) t = 30 and 500 ns for the second repeat simulation. (*C*) Binding site occupancies for GM3 headgroups within 6 Å of sites 1 and 2 (see parts *E* and *F*) over the 2 × 500-ns simulations. Site occupancies were normalized from white (no GM3 headgroups atoms within 6 Å) to black (the maximal number of GM3 headgroup atoms within 6 Å of the site). (*D*) Snapshots of $GCGR_{apo}$ from the two atomistic simulations at time points corresponding to the arrows embedded in a mixed lipid membrane containing 10% GM3 (shown in *red/orange*). The ECD-TMD angles are marked. (*E*) The GCGR at 500 ns showing a GM3 molecule bound to site 1 on the ECD (residues in *pink*; see text for further details) and also indicating the location of site 2 (in *blue*). (*F*) Zoomed-in view of GM3 bound at site 1, indicating the key residues involved in the protein-lipid interactions. To see this figure in color, go online.

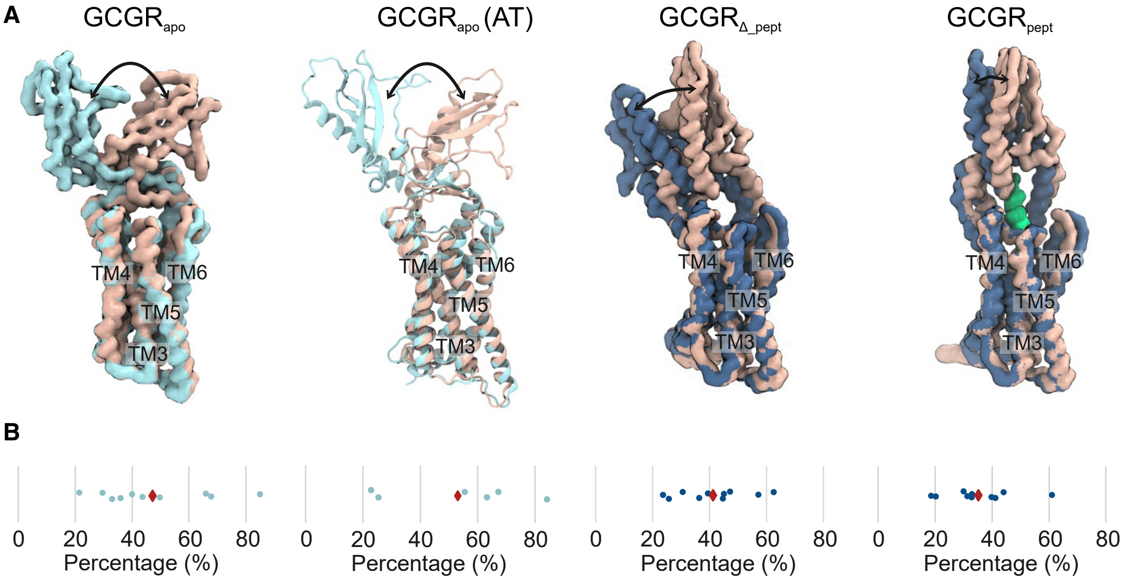

FIGURE 4 GCGR conformational dynamics. Principal component analysis of GCGR dynamics was performed for CG simulations (10 × 10 μs) of $GCGR_{apo}$, $GCGR_{pept}$, and $GCGR_{\Delta\_pept}$ and for atomistic simulations (6 × 500 ns) of $GCGR_{apo}$, all embedded in bilayers containing 10% GM3. Each replicate was equilibrated independently. (*A*) Representative examples of motions corresponding to the first principal component, colored according to when the ECD is furthest from the bilayer (*ochre*) or when the ECD opens toward the bilayer (*light blue*: $GCGR_{apo}$, *dark blue*: $GCGR_{pept}$/$GCGR_{\Delta\_pept}$). NNC1702 peptide is colored lime. Movement of the ECD is indicated by arrows. (*B*) The percentages of motion represented by the first eigenvalue for each simulation replicate are shown as blue circles, with the mean percentage for each simulation shown as a red diamond. To see this figure in color, go online.

therefore could allosterically modulate the function of GCGR via, e.g., altering the rate of ligand recruitment. In our simulations, the hinge region that connects ECD and TMD is flexible, allowing the ECD to adopt different orientations. This flexibility agreed well with the observation of varied ECD conformations among class B1 GPCRs (Fig. S4), and the importance of ECD dynamics has been stressed in a number of studies (2,13,65,66). Our simulations further reveal that different conformations of the ECD have different dynamic behavior, which may have functional relevance, e.g., large-scale movements between closed and open states of the GCGR may facilitate peptide ligand recruitment to the receptor.

GM3 interactions with GCGR

Given the observation of close localization of GM3 around GCGR in the extracellular leaflet, we postulated that GM3 interactions may have a modulatory effect on ECD dynamics. Indeed, a number of recent studies suggest lipids may play a role in the regulation of GPCRs and in coupling to downstream signaling components (14,16,17). We used protein-lipid contact mapping to assess the interaction profiles of GM3 and PIP_2 with GCGR as a first step toward understanding how these two key lipids might influence GCGR behavior. GM3 headgroup interactions with the GCGR TMD were conserved across CG simulations in bilayers containing different concentrations of GM3, interacting with ECD loops ECL1–3 and the extracellular regions of TM1–7 (Fig. 5; the convergence of estimates of these interactions between simulation replicates is addressed in Fig. S6). The GM3 interactions sites seen in atomistic simulations were similar to those in CG, even though less sampling has led to sparser interactions. This good agreement indicates that the observed interactions are consistent between the different simulation granularities.

The GM3 interaction profiles revealed conformational dependence when comparing between the ECDs of $GCGR_{apo}$, $GCGR_{pept}$, and $GCGR_{\Delta\_pept}$ (Fig. 5; Fig. S5 B). Thus, $GCGR_{apo}$ and $GCGR_{\Delta\_pept}$ both form interactions of GM3 with the α_1-helix of the ECD (Q27-K37), with the β_3-β_4 loop, and with the ECD-TMD Stalk linker (P86-Q131) because of the proximity of these regions to the bilayer. These interactions overlay with site 1 discussed above, at which GM3 binding correlates with ECD opening. The interaction fingerprints of $GCGR_{apo}$ at 5 and 10% GM3 are similar, interacting with L38-L85 in addition to ECD regions proximal to the bilayer (Figs. 2 *D* and 5). In the $GCGR_{apo}$ crystal structure, these residues are located 0.5–3.5 nm beyond the terminal GM3 sugar moiety, and therefore contacts can only occur when the ECD opens toward the bilayer. For $GCGR_{pept}$ the ECD-GM3 contacts are limited because of restriction of receptor conformation by the bound peptide. GM3 contacts are confined to the α_1-helix and the Stalk region, within the width of the GM3 glycan layer. When we removed the peptide agonist from our simulations, we are able to recover GM3 contacts with D63-D124, including extended interactions with G109-D124 in

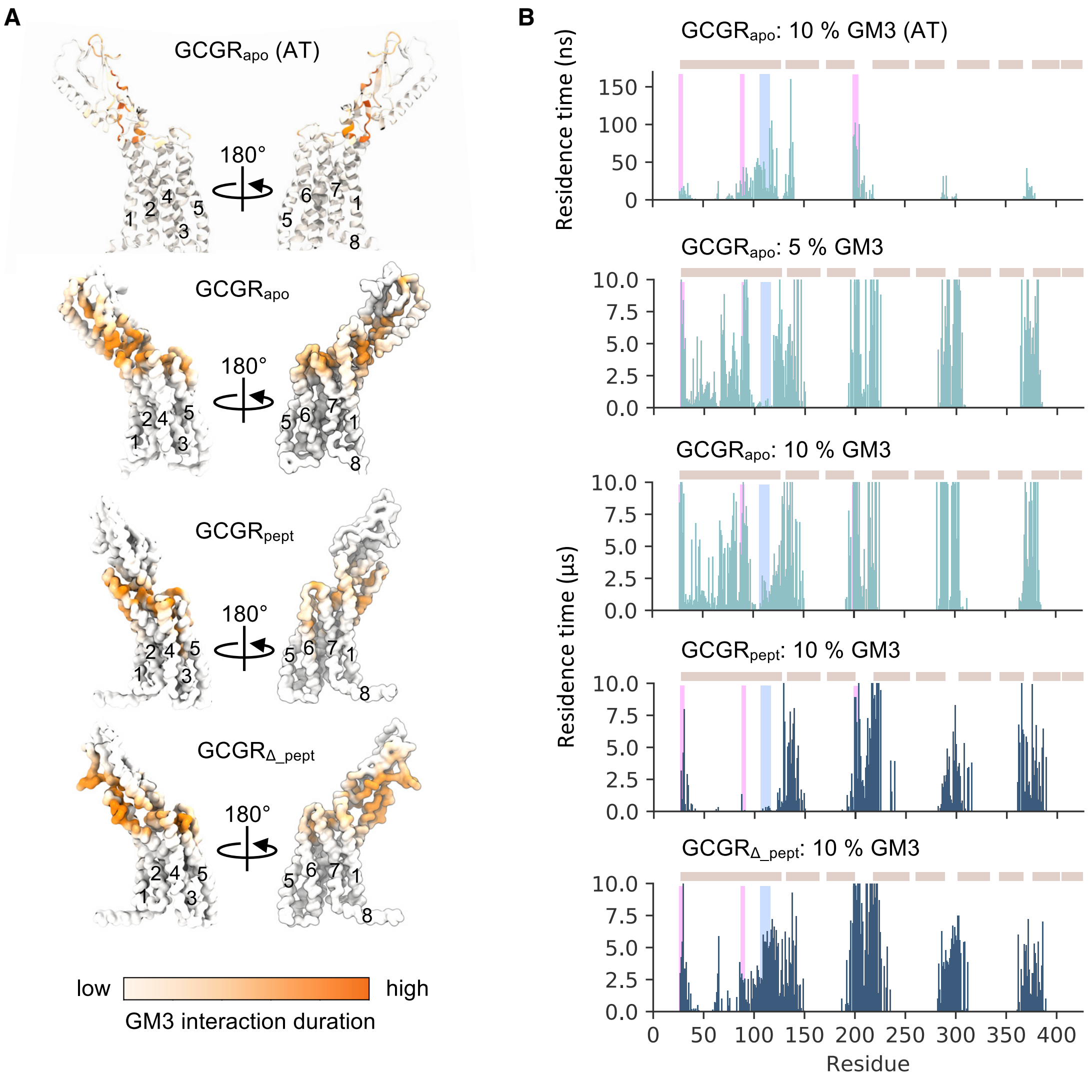

FIGURE 5 Interactions of GCGR with GM3. (*A*) Comparison of GM3 interactions in bilayers containing 10% GM3 mapped onto the structure of $GCGR_{apo}$ from CG and atomistic (AT) simulations and $GCGR_{pept}$/$GCGR_{\Delta\_pept}$ from CG simulations. Contacts are colored from regions of low (*white*) to high (*orange*) mean residence times. (*B*) GM3 headgroup interaction profiles with $GCGR_{apo}$ from atomistic simulations (6 × 0.5 μs) and $GCGR_{apo}$, $GCGR_{pept}$, and $GCGR_{\Delta\_pept}$ in CG simulations (10 × 10 μs) in mixed lipid bilayers containing 0–10% GM3. GM3 residence times were calculated using a 0.55- and 1.0-nm dual cutoff scheme in CG simulations or 0.35- and 0.55 -nm in atomistic simulations. Positions of ECD interaction site 1 (*pink*) and site 2 (*cyan*) (see Fig. 3) are shown as rectangles on the contact plot. The position of the ECD of TM1-7 and of H8 are shown above the contact profile as ochre rectangles. Each replicate was equilibrated independently to aid protein-lipid contact sampling. To see this figure in color, go online.

the $GCGR_{\Delta\_pept}$ simulations. This suggests that the different ECD conformations do not restrict the ability for the ECD to contact the bilayer but peptide binding does do so.

GM3 binding sites were seen to be more extended than, e.g., PIP_2 binding sites (see below), in part because of the size and flexibility of the ganglioside headgroup. A range of nonpolar, polar, and charged residues interacted with the glycan headgroup (Fig. S7). This diversity of GM3 interactions suggests that they may be quite malleable and hence that the observed modulatory effect of GM3 interactions on ECD conformational dynamics could be shared with other class B GPCRs. Although relatively little is known about glycosphingolipid interactions with membrane proteins, given the large number of hydroxyl groups present in the glycosphingolipid glycans and the increased size of their headgroup compared with other lipids, it is perhaps unsurprising that interactions are less electrostatically driven than one might assume. This is supported by our observations from atomistic simulations that show small hydrophobic and polar amino acid side chains in addition to cationic side chains interacting with GM3 (Fig. 3 *F*). We also performed CG simulations of $GCGR_{apo}$ embedded in a mixed lipid mixture containing GM1 instead of GM3 (Table 1). GM1 was observed to localize around $GCGR_{apo}$ and contact the ECD in a similar manner observed for GM3 (Fig. S8 A). Mapping both GM3 and GM1 headgroup interactions with

GCGR$_{apo}$ onto the protein surface (Fig. S8 B) revealed substantial overlap between GM3 and GM1 interactions. However, GM1 headgroup interactions extended further from the bilayer surface, as expected given its larger size. This suggests GCGR interactions with glycosphingolipids may not be limited to GM3. On the basis of our site 1 pose identified in atomistic simulations (Fig. 3 *F*) and of this per-residue contact data (Fig. S7), we suggest that residues L32, S203, or K35 may be suitable candidates for mutations to probe the role of GM3 interactions with GCGR.

Differences between PIP$_2$ interactions with GCGR and class A receptors

PIP$_2$ has recently emerged as a potential regulator of GPCR state and protein-coupling selectivity (14,16). Analysis of PIP$_2$ interactions revealed a conserved interaction fingerprint for all CG simulations of GCGR in a mixed lipid membrane (Fig. 6 *A*; Figs. S5 C and S6). PIP$_2$ molecules bound to sites defined by TM1/ICL1/TM2/TM4, by TM5/ICL3, and by TM6/TM7 and H8, interacting predominantly via their anionic headgroups with cationic (ARG and LYS) residues or via the hydroxyl groups of SER and THR (Fig. 6 *B*). The PIP$_2$ contact profile at each interaction site is narrower than for GM3, while residence times for PIP$_2$ interactions are generally lower than for GM3 (compare Figs. 5 *B* and 6 *A*). For GCGR$_{apo}$, PIP$_2$ residence times were longest for the TM1/ICL1/TM2/TM4, TM5/ICL3, and TM6/TM7 sites. GCGR$_{pept}$/GCGR$_{\Delta_pept}$ showed reduced PIP$_2$ residence times at TM1/ICL1/TM2/TM4, TM5/ICL3, and TM6/TM7 and enhanced PIP$_2$ interaction with H8 compared with GCGR$_{apo}$, suggesting conformation-specific differences in PIP$_2$ binding, which may be implicated in allosteric regulation of the receptor.

We compared the interaction profile of GCGR to the prototypical class A receptor A$_{2A}$ (28). There was agreement between the class A and class B1 receptors for PIP$_2$ binding to TM1/ICL1/TM2/TM4 and TM6/7. In particular, the

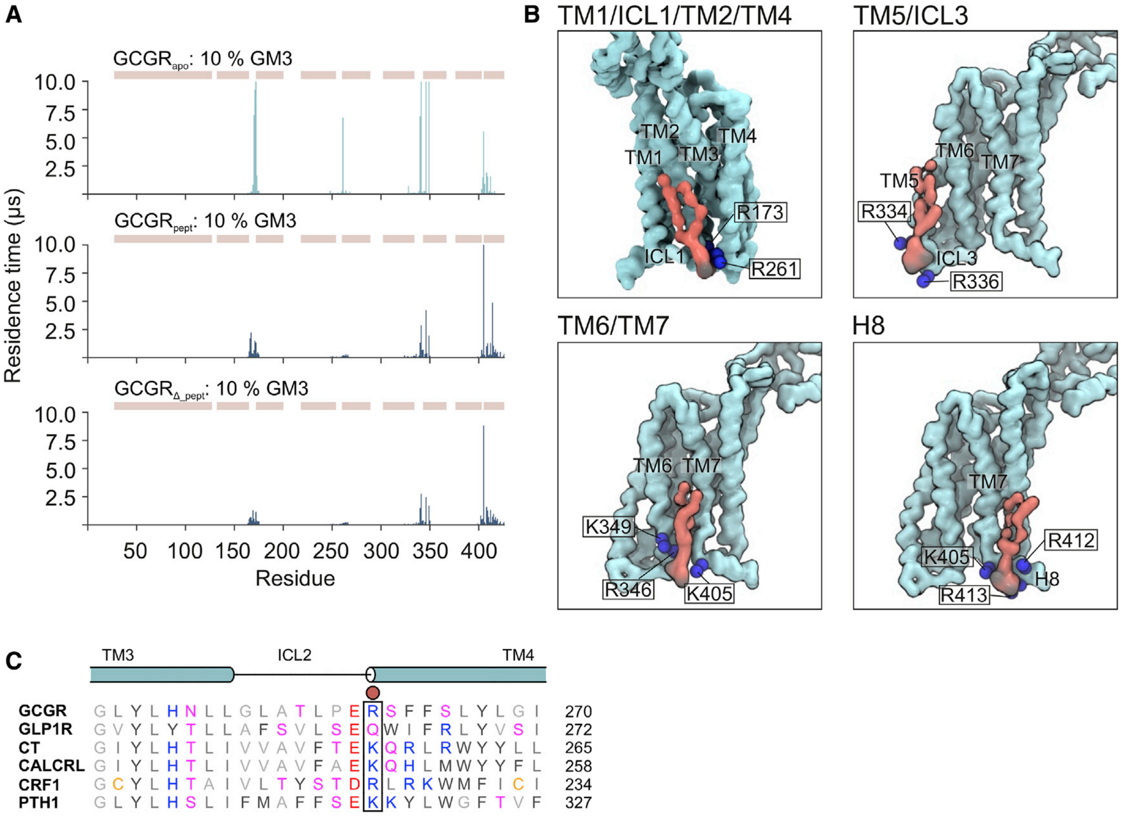

FIGURE 6 PIP$_2$ interactions with GCGR. (*A*) PIP$_2$ interaction profiles with GCGR$_{apo}$, GCGR$_{pept}$, and GCGR$_{\Delta_pept}$ in CG simulations (10×10 μs) in mixed lipid bilayers containing 0–10% GM3. PIP$_2$ headgroup residence times were calculated using a 0.55- and 1.0-nm dual cutoff scheme. The position of the ECD, TM1-7, and H8 regions are shown above the contact profile as ochre rectangles. Each replicate was equilibrated independently to aid protein-lipid contact sampling. (*B*) PIP$_2$ binding poses identified in CG simulations. PIP$_2$ (*red*) is shown bound to GCGR$_{apo}$ (*light blue*). PIP$_2$ phosphate groups are colored black, and K and R residues are shown as blue spheres. (*C*) Structure-based sequence alignment of class B1 GPCRs showing conservation of the basic R/K residue at the N-terminus of TM4. A red circle shows the position of GCGR$_{apo}$ R261 (see *B*), which contributes to binding of PIP$_2$ at the TM1/ICL1/TM2/TM4 site. Structure-based sequence alignment was performed on GPCRdb.org using the human calcitonin (CT), calcitonin receptor-like (CALCRL), corticotropin-releasing factor 1 (CRF1), glucagon-like peptide-1 (GLP1R), glucagon (GCGR), and parathyroid hormone-1 (PTH1) receptors, with manual adjustment based on the position of helices observed in structures. To see this figure in color, go online.

interaction between the anionic PIP_2 headgroup and a basic residue at the N-terminus of the TM4 helix (see Fig. 6, *B* and *C*) is conserved across both class A and class B1 GPCRs and is seen in the structure of PIP_2 bound to the NTS1R/β-arrestin-1 complex (16). However, in contrast to the A_{2A} receptor, PIP_2 binding was not observed in the vicinity of TM3 or ICL2 of GCGR. Interactions of PIP_2 with the A_{2A} receptor at TM3/ICL2/TM4 have been suggested to enhance interaction with a mini-Gs-protein, acting as a "glue" between the receptor and the G-protein (28). A lack of PIP_2 interactions at this site for GCGR may indicate differences in the influence of the anionic headgroups on G-protein coupling that is less dependent on PIP_2 bridging interactions between the two proteins. Furthermore, a structure-based sequence alignment of class B1 GPCRs showed conservation of positive residues at ICL1 (R/$K^{12.48}$), TM2 ($R^{2.46}$), TM4 (R/$K^{4.39}$), TM5 ($K^{5.64}$, R/$K^{5.66}$), TM6 (R/$K^{6.37}$, R/$K^{6.40}$), and H8 (R/$K^{8.55}$, R/$K^{8.56}$) but not at ICL2 or the intracellular end of TM3, suggesting that a lack of PIP_2 binding at TM3/ICL2 may be a conserved feature across class B1 GPCRs (Fig. 6 *C*; Fig. S8). This lack of interaction at TM3/ICL2 suggests that the involvement of PIP_2 in recruitment of signaling partners in class B1 GPCRs may be different from that in class A GPCRs.

CONCLUSIONS

MD simulations starting from a number of distinct GCGR conformations have been used to explore the relationship between lipid interactions and the conformational dynamics of the receptor (Fig. 7). Two key lipid species, GM3 in the extracellular leaflet and PIP_2 in the intracellular leaflet, formed contacts with the GCGR. By probing GM3 interactions in different GCGR conformations and in membranes of different GM3 concentrations, we revealed that the binding of GM3 to different parts of GCGR led to generation of different ECD conformations. The multiplicity of ECD conformations could prepare GCGR for the various tasks along its signaling pathways. Evidence from cross-linking, hydrogen-deuterium exchange, MD, and mutagenesis studies suggest that an inactive state of GCGR is favored by interactions of the ECD with ECL1 or ECL3 (5,10,69). The observation that the binding of GM3 to site 1 led to opening of GCGR ECD in our simulations suggested that increasing the GM3 concentration in the local environment could shift the receptor toward active states. The varied concentrations of glycosphingolipids in different microdomains of membranes and cellular compartments could therefore contribute to tuning of the signaling properties of GCGR. It is tempting to speculate that changes in lipid metabolism (as a result of dietary intake (70) and/or pharmacological intervention (71)) may lead to changes in lipid rafts. This in turn may affect the relative proportions of the insulin and GCGRs localized within raft and nonraft membrane microdomains. GM3 has been observed to promote insulin receptor removal from rafts and decrease insulin signaling (72,73). It is not unreasonable to suggest that GM3 may also play a role in regulation of glucagon signaling and therefore of the overall insulin/glucagon signaling ratio. However, additional experimental studies would be needed to test this.

In addition to GM3, we identified four PIP_2 binding sites on GCGR that showed major differences around TM3/ICL2 when compared with PIP_2 interactions with class A GPCR A2aR. This could indicate distinct mechanisms of engaging with G-protein and β-arrestin partners whereby class B1 receptors may have a different dependence on lipid mediatory interactions to bridge the receptor-G-protein interface compared with class A GPCRs. This may be important for differentiation of receptor signaling and recycling times, potentially contributing to the observation that class B1 GPCRs have sustained signaling (e.g., (74)) compared with most class A receptors, postulated to result from enhanced interactions with β-arrestins, which may contribute to formation of GPCR G-protein/β-arrestin hybrid complexes (75).

We recognize that one area for future research will be the effects of lipid headgroup charge states and possible interactions with counterions on the interactions of lipids with the GCGR and related proteins. There have been a number of studies of the likely importance of such effects for PIP_2 (76,77); for gangliosides, the situation is less clear, although a number of studies (e.g., (78,79)) suggest interactions with

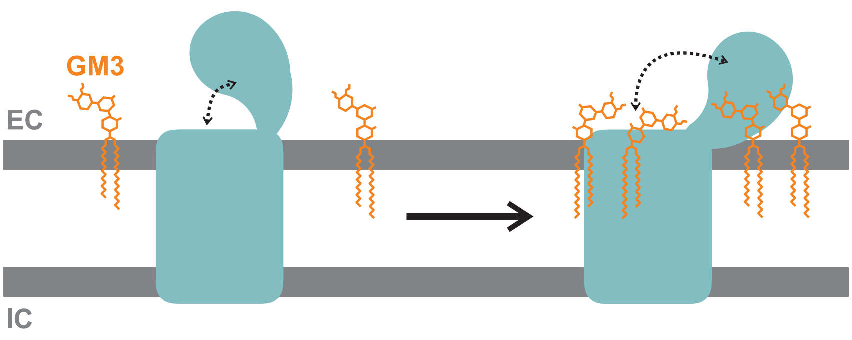

FIGURE 7 GM3 binding to GCGR promotes ECD opening. Schematic overview of the effect of GM3 (*orange*) on the behavior of GCGR (*light blue*) when devoid of peptide ligands. GM3 binds the receptor TMD and ECD. GM3 binding to the ECD causes conformational modulation of GCGR such that the ECD moves toward the membrane, exposing the peptide ligand binding pocket. The positions of extracellular (EC) and intracellular (IC) leaflets are marked. To see this figure in color, go online.

counterions would merit further investigation, especially in the context of likely self-association of gangliosides in membranes (68,80). In addition to understanding better the physicochemical properties of more complex mixtures of lipids, it will be important to integrate these with ongoing advances in lipidome-based models of the cell membrane environment (32). There also remains a need for more experimental data on receptor/ganglioside interactions, both via mutational studies, and as a consequence of, e.g., improvements in cryo-EM studies, which have recently been shown to be able to resolve interactions of receptors and channels with PIP_2 (16,81). It will also be of interest to conduct larger-scale membrane simulations to explore whether the interactions of gangliosides with GCGRs can result in local membrane deformations and/or changes in mechanical properties.

Overall, our simulations provide structural insight into the behavior of GCGR in mixed lipid asymmetric bilayers that mimic aspects of the composition of the plasma membrane. We observe modulation of ECD dynamics by the glycosphingolipid GM3, providing an additional layer of complexity to previous observations of GCGR ECD dynamics around the hinge region (10,12). We observe differences in PIP_2 binding to GCGR compared with class A receptors, which may have functional implications for signaling properties across the class B1 family. Thus, these data provide a structural basis for further functional investigation of the role of glycosphingolipids and phosphatidylinositols in modulating GCGR signaling, localization, and protein coupling in vivo.

SUPPORTING MATERIAL

Supporting Material can be found online at https://doi.org/10.1016/j.bpj.2020.06.009.

AUTHOR CONTRIBUTIONS

M.S.P.S., W.S., and T.B.A. conceptualized the project. W.S. and T.B.A. conducted the simulations and analyzed the data. M.S.P.S., W.S., and T.B.A. wrote the manuscript.

ACKNOWLEDGMENTS

Research in the M.S.P.S. group is supported by Wellcome, BBSRC, and EPSRC. W.S. acknowledges support from the Newton International Fellowship. T.B.A. acknowledges support from Wellcome. This project made use of time on ARCHER via the HECBioSim, supported by EPSRC.

REFERENCES

1. Culhane, K. J., Y. Liu, …, E. C. Y. Yan. 2015. Transmembrane signal transduction by peptide hormones via family B G protein-coupled receptors. *Front. Pharmacol.* 6:264.
2. de Graaf, C., G. Song, …, R. C. Stevens. 2017. Extending the structural view of class B GPCRs. *Trends Biochem. Sci.* 42:946–960.
3. Karageorgos, V., M. Venihaki, …, G. Liapakis. 2018. Current understanding of the structure and function of family B GPCRs to design novel drugs. *Hormones (Athens).* 17:45–59.
4. Parthier, C., S. Reedtz-Runge, …, M. T. Stubbs. 2009. Passing the baton in class B GPCRs: peptide hormone activation via helix induction? *Trends Biochem. Sci.* 34:303–310.
5. Zhao, L. H., Y. Yin, …, H. E. Xu. 2016. Differential requirement of the extracellular domain in activation of class B G protein-coupled receptors. *J. Biol. Chem.* 291:15119–15130.
6. Ramnanan, C. J., D. S. Edgerton, …, A. D. Cherrington. 2011. Physiologic action of glucagon on liver glucose metabolism. *Diabetes Obes. Metab.* 13 (*Suppl 1*):118–125.
7. Holst, J. J., N. J. Wewer Albrechtsen, …, F. K. Knop. 2017. Glucagon and amino acids are linked in a mutual feedback cycle: the liver-α-cell axis. *Diabetes.* 66:235–240.
8. Galsgaard, K. D., J. Pedersen, …, N. J. Wewer Albrechtsen. 2019. Glucagon receptor signaling and lipid metabolism. *Front. Physiol.* 10:413.
9. NCD Risk Factor Collaboration (NCD-RisC). 2016. Worldwide trends in diabetes since 1980: a pooled analysis of 751 population-based studies with 4.4 million participants. *Lancet.* 387:1513–1530.
10. Zhang, H., A. Qiao, …, B. Wu. 2017. Structure of the full-length glucagon class B G-protein-coupled receptor. *Nature.* 546:259–264.
11. Zhang, H., A. Qiao, …, B. Wu. 2018. Structure of the glucagon receptor in complex with a glucagon analogue. *Nature.* 553:106–110.
12. Yang, L., D. Yang, …, H. Jiang. 2015. Conformational states of the full-length glucagon receptor. *Nat. Commun.* 6:7859.
13. Wu, F., L. Yang, …, R. C. Stevens. 2020. Full-length human GLP-1 receptor structure without orthosteric ligands. *Nat. Commun.* 11:1272.
14. Yen, H. Y., K. K. Hoi, …, C. V. Robinson. 2018. PtdIns(4,5)P_2 stabilizes active states of GPCRs and enhances selectivity of G-protein coupling. *Nature.* 559:423–427.
15. Duncan, A. L., W. Song, and M. S. P. Sansom. 2020. Lipid-dependent regulation of ion channels and G protein-coupled receptors: insights from structures and simulations. *Annu. Rev. Pharmacol. Toxicol.* 60:31–50.
16. Huang, W., M. Masureel, …, B. K. Kobilka. 2020. Structure of the neurotensin receptor 1 in complex with β-arrestin 1. *Nature.* 579:303–308.
17. Dawaliby, R., C. Trubbia, …, C. Govaerts. 2016. Allosteric regulation of G protein-coupled receptor activity by phospholipids. *Nat. Chem. Biol.* 12:35–39.
18. Bruzzese, A., C. Gil, …, J. Giraldo. 2018. Structural insights into positive and negative allosteric regulation of a G protein-coupled receptor through protein-lipid interactions. *Sci. Rep.* 8:4456.
19. Shan, J., G. Khelashvili, …, H. Weinstein. 2012. Ligand-dependent conformations and dynamics of the serotonin 5-HT($_{2A}$) receptor determine its activation and membrane-driven oligomerization properties. *PLoS Comput. Biol.* 8:e1002473.
20. Grossfield, A., S. E. Feller, and M. C. Pitman. 2006. A role for direct interactions in the modulation of rhodopsin by omega-3 polyunsaturated lipids. *Proc. Natl. Acad. Sci. USA.* 103:4888–4893.
21. Hedger, G., and M. S. P. Sansom. 2016. Lipid interaction sites on channels, transporters and receptors: recent insights from molecular dynamics simulations. *Biochim. Biophys. Acta.* 1858:2390–2400.
22. Sengupta, D., X. Prasanna, …, A. Chattopadhyay. 2018. Exploring GPCR-lipid interactions by molecular dynamics simulations: excitements, challenges, and the way forward. *J. Phys. Chem. B.* 122:5727–5737.
23. Corradi, V., B. I. Sejdiu, …, D. P. Tieleman. 2019. Emerging diversity in lipid-protein interactions. *Chem. Rev.* 119:5775–5848.
24. Hanson, M. A., V. Cherezov, …, R. C. Stevens. 2008. A specific cholesterol binding site is established by the 2.8 A structure of the human β2-adrenergic receptor. *Structure.* 16:897–905.

25. Cang, X., Y. Du, …, H. Jiang. 2013. Mapping the functional binding sites of cholesterol in β2-adrenergic receptor by long-time molecular dynamics simulations. *J. Phys. Chem. B.* 117:1085–1094.

26. Prasanna, X., A. Chattopadhyay, and D. Sengupta. 2014. Cholesterol modulates the dimer interface of the β_2-adrenergic receptor via cholesterol occupancy sites. *Biophys. J.* 106:1290–1300.

27. Manna, M., M. Niemelä, …, I. Vattulainen. 2016. Mechanism of allosteric regulation of β_2-adrenergic receptor by cholesterol. *eLife.* 5:e18432.

28. Song, W., H. Y. Yen, …, M. S. P. Sansom. 2019. State-dependent lipid interactions with the A2a receptor revealed by MD simulations using in vivo-mimetic membranes. *Structure.* 27:392–403.e3.

29. Siu, F. Y., M. He, …, R. C. Stevens. 2013. Structure of the human glucagon class B G-protein-coupled receptor. *Nature.* 499:444–449.

30. Li, Y., J. Sun, …, J. Lin. 2016. Activation and conformational dynamics of a class B G-protein-coupled glucagon receptor. *Phys. Chem. Chem. Phys.* 18:12642–12650.

31. Marrink, S. J., V. Corradi, …, M. S. P. Sansom. 2019. Computational modeling of realistic cell membranes. *Chem. Rev.* 119:6184–6226.

32. Lorent, J. H., K. R. Levental, …, I. Levental. 2020. Plasma membranes are asymmetric in lipid unsaturation, packing and protein shape. *Nat. Chem. Biol.* 16:644–652.

33. Coskun, Ü., M. Grzybek, …, K. Simons. 2011. Regulation of human EGF receptor by lipids. *Proc. Natl. Acad. Sci. USA.* 108:9044–9048.

34. Hedger, G., D. Shorthouse, …, M. S. Sansom. 2016. Free energy landscape of lipid interactions with regulatory binding sites on the transmembrane domain of the EGF receptor. *J. Phys. Chem. B.* 120:8154–8163.

35. Fiser, A., and A. Šali. 2003. Modeller: generation and refinement of homology-based protein structure models. *Methods Enzymol.* 374:461–491.

36. de Jong, D. H., G. Singh, …, S. J. Marrink. 2013. Improved parameters for the Martini coarse-grained protein force field. *J. Chem. Theory Comput.* 9:687–697.

37. Periole, X., M. Cavalli, …, M. A. Ceruso. 2009. Combining an elastic network with a coarse-grained molecular force field: structure, dynamics, and intermolecular recognition. *J. Chem. Theory Comput.* 5:2531–2543.

38. Wassenaar, T. A., H. I. Ingólfsson, …, S. J. Marrink. 2015. Computational lipidomics with *insane*: a versatile tool for generating custom membranes for molecular simulations. *J. Chem. Theory Comput.* 11:2144–2155.

39. Melo, M. N., H. I. Ingólfsson, and S. J. Marrink. 2015. Parameters for Martini sterols and hopanoids based on a virtual-site description. *J. Chem. Phys.* 143:243152.

40. Marrink, S. J., H. J. Risselada, …, A. H. de Vries. 2007. The MARTINI force field: coarse grained model for biomolecular simulations. *J. Phys. Chem. B.* 111:7812–7824.

41. Monticelli, L., S. K. Kandasamy, …, S. J. Marrink. 2008. The MARTINI coarse-grained force field: extension to proteins. *J. Chem. Theory Comput.* 4:819–834.

42. Bussi, G., D. Donadio, and M. Parrinello. 2007. Canonical sampling through velocity rescaling. *J. Chem. Phys.* 126:014101.

43. Parrinello, M., and A. Rahman. 1981. Polymorphic transitions in single-crystals - a new molecular-dynamics method. *J. Appl. Phys.* 52:7182–7190.

44. Hess, B., H. Bekker, …, J. G. E. M. Fraaije. 1997. LINCS: a linear constraint solver for molecular simulations. *J. Comput. Chem.* 18:1463–1472.

45. Jo, S., J. B. Lim, …, W. Im. 2009. CHARMM-GUI membrane builder for mixed bilayers and its application to yeast membranes. *Biophys. J.* 97:50–58.

46. Wu, E. L., X. Cheng, …, W. Im. 2014. CHARMM-GUI membrane builder toward realistic biological membrane simulations. *J. Comput. Chem.* 35:1997–2004.

47. Lee, J., D. S. Patel, …, W. Im. 2019. CHARMM-GUI membrane builder for complex biological membrane simulations with glycolipids and lipoglycans. *J. Chem. Theory Comput.* 15:775–786.

48. Wolf, M. G., M. Hoefling, …, G. Groenhof. 2010. g_membed: efficient insertion of a membrane protein into an equilibrated lipid bilayer with minimal perturbation. *J. Comput. Chem.* 31:2169–2174.

49. Jorgensen, W. L., J. Chandresekhar, …, M. L. Klein. 1983. Comparison of simple potential functions for simulating liquid water. *J. Chem. Phys.* 79:926–935.

50. Huang, J., and A. D. MacKerell, Jr. 2013. CHARMM36 all-atom additive protein force field: validation based on comparison to NMR data. *J. Comput. Chem.* 34:2135–2145.

51. Darden, T., D. York, and L. Pedersen. 1993. Particle mesh Ewald - an N.log(N) method for Ewald sums in large systems. *J. Chem. Phys.* 98:10089–10092.

52. Nose, S. 1984. A molecular dynamics method for simulations in the canonical ensemble. *Mol. Phys.* 52:255–268.

53. Hoover, W. G. 1985. Canonical dynamics: equilibrium phase-space distributions. *Phys. Rev. A Gen. Phys.* 31:1695–1697.

54. Humphrey, W., A. Dalke, and K. Schulten. 1996. VMD: visual molecular dynamics. *J. Mol. Graph.* 14:33–38, 27–28.

55. Schrodinger LLC. 2010. The PyMOL molecular graphics system, version 1.3r1.

56. Sampaio, J. L., M. J. Gerl, …, A. Shevchenko. 2011. Membrane lipidome of an epithelial cell line. *Proc. Natl. Acad. Sci. USA.* 108:1903–1907.

57. Prasanna, X., M. Jafurulla, …, A. Chattopadhyay. 2016. The ganglioside GM1 interacts with the serotonin$_{1A}$ receptor via the sphingolipid binding domain. *Biochim. Biophys. Acta.* 1858:2818–2826.

58. Hedger, G., H. Koldsø, …, M. S. P. Sansom. 2019. Cholesterol interaction sites on the transmembrane domain of the hedgehog signal transducer and Class F G protein-coupled receptor smoothened. *Structure.* 27:549–559.e2.

59. Jazayeri, A., M. Rappas, …, F. H. Marshall. 2017. Crystal structure of the GLP-1 receptor bound to a peptide agonist. *Nature.* 546:254–258.

60. Liang, Y. L., M. Khoshouei, …, D. Wootten. 2018. Phase-plate cryo-EM structure of a biased agonist-bound human GLP-1 receptor-Gs complex. *Nature.* 555:121–125.

61. Zhang, Y., B. Sun, …, G. Skiniotis. 2017. Cryo-EM structure of the activated GLP-1 receptor in complex with a G protein. *Nature.* 546:248–253.

62. Liang, Y. L., M. Khoshouei, …, P. M. Sexton. 2018. Cryo-EM structure of the active, G $_s$-protein complexed, human CGRP receptor. *Nature.* 561:492–497.

63. Ehrenmann, J., J. Schöppe, …, A. Plückthun. 2018. High-resolution crystal structure of parathyroid hormone 1 receptor in complex with a peptide agonist. *Nat. Struct. Mol. Biol.* 25:1086–1092.

64. Zhao, L. H., S. Ma, …, Y. Zhang. 2019. Structure and dynamics of the active human parathyroid hormone receptor-1. *Science.* 364:148–153.

65. Liang, Y. L., M. J. Belousoff, …, D. Wootten. 2020. Toward a structural understanding of class B GPCR peptide binding and activation. *Mol. Cell.* 77:656–668.e5.

66. Ma, S., Q. Shen, …, H. E. Xu. 2020. Molecular basis for hormone recognition and activation of corticotropin-releasing factor receptors. *Mol. Cell.* 77:669–680.e4.

67. van Meer, G., and A. I. P. M. de Kroon. 2011. Lipid map of the mammalian cell. *J. Cell Sci.* 124:5–8.

68. Koldsø, H., D. Shorthouse, …, M. S. P. Sansom. 2014. Lipid clustering correlates with membrane curvature as revealed by molecular simulations of complex lipid bilayers. *PLoS Comput. Biol.* 10:e1003911.

69. Koth, C. M., J. M. Murray, …, B. B. Allan. 2012. Molecular basis for negative regulation of the glucagon receptor. *Proc. Natl. Acad. Sci. USA.* 109:14393–14398.

70. Metcalfe, L. K., G. C. Smith, and N. Turner. 2018. Defining lipid mediators of insulin resistance - controversies and challenges. *J. Mol. Endocrinol.* 62:R65–R82.

71. Shrivastava, S., T. J. Pucadyil, …, A. Chattopadhyay. 2010. Chronic cholesterol depletion using statin impairs the function and dynamics of human serotonin(1A) receptors. *Biochemistry.* 49:5426–5435.

72. Yamashita, T., A. Hashiramoto, …, R. L. Proia. 2003. Enhanced insulin sensitivity in mice lacking ganglioside GM3. *Proc. Natl. Acad. Sci. USA.* 100:3445–3449.

73. Kabayama, K., T. Sato, …, J. Inokuchi. 2005. TNFalpha-induced insulin resistance in adipocytes as a membrane microdomain disorder: involvement of ganglioside GM3. *Glycobiology.* 15:21–29.

74. Ferrandon, S., T. N. Feinstein, …, J. P. Vilardaga. 2009. Sustained cyclic AMP production by parathyroid hormone receptor endocytosis. *Nat. Chem. Biol.* 5:734–742.

75. Thomsen, A. R. B., B. Plouffe, …, R. J. Lefkowitz. 2016. GPCR-G protein-β-arrestin super-complex mediates sustained G protein signaling. *Cell.* 166:907–919.

76. Slochower, D. R., P. J. Huwe, …, P. A. Janmey. 2013. Quantum and all-atom molecular dynamics simulations of protonation and divalent ion binding to phosphatidylinositol 4,5-bisphosphate (PIP_2). *J. Phys. Chem. B.* 117:8322–8329.

77. Bilkova, E., R. Pleskot, …, Ü. Coskun. 2017. Calcium directly regulates phosphatidylinositol 4,5-bisphosphate headgroup conformation and recognition. *J. Am. Chem. Soc.* 139:4019–4024.

78. Rodgers, J. C., and P. S. Portoghese. 1994. Molecular modeling of the conformational and sodium ion binding properties of the oligosaccharide component of ganglioside GM1. *Biopolymers.* 34:1311–1326.

79. Del Favero, E., P. Brocca, …, L. Cantu'. 2014. Optimizing the crowding strategy: sugar-based ionic micelles in the dilute-to-condensed regime. *Langmuir.* 30:9157–9164.

80. Gu, R. X., H. I. Ingólfsson, …, D. P. Tieleman. 2017. Ganglioside-lipid and ganglioside-protein interactions revealed by coarse-grained and atomistic molecular dynamics simulations. *J. Phys. Chem. B.* 121:3262–3275.

81. Laverty, D., R. Desai, …, A. R. Aricescu. 2019. Cryo-EM structure of the human α1β3γ2 $GABA_A$ receptor in a lipid bilayer. *Nature.* 565:516–520.

Biophysical Journal, Volume *119*

Supplemental Information

The Glycosphingolipid GM3 Modulates Conformational Dynamics of the Glucagon Receptor

T. Bertie Ansell, Wanling Song, and Mark S.P. Sansom

Supporting Material

Lipid parameters GROMACS *.*itp* files for PIP2 and GM3 are provided as data files.

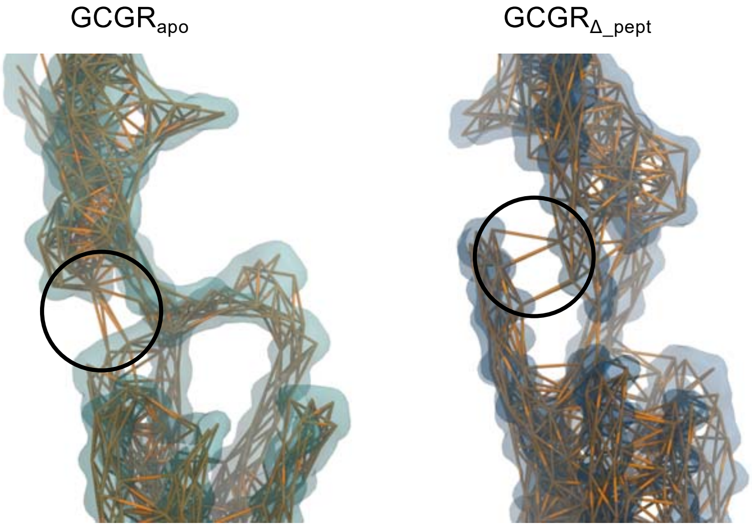

Supplementary Figure S1: Elastic network restraints in CG GCGR structures. Structures of $GCGR_{apo}$ (5XEZ) and $GCGR_{\Delta\_pept}$ (5YQZ) coarse-grained using the MARTINI 2.2 forcefield with an ElNeDyn elastic network (spring force constant = 500 $kJ.mol^{-1}.nm^{-2}$, cut-off = 0.9 nm). The elastic network was visualised using the *cg_bonds.tcl* script with a 0.9 nm cut-off (cgmartini.nl/index.php/tools2/visualization). Circles indicate elastic network restraints connecting the ECD and TMD regions of $GCGR_{apo}$ and $GCGR_{\Delta\_pept}$.

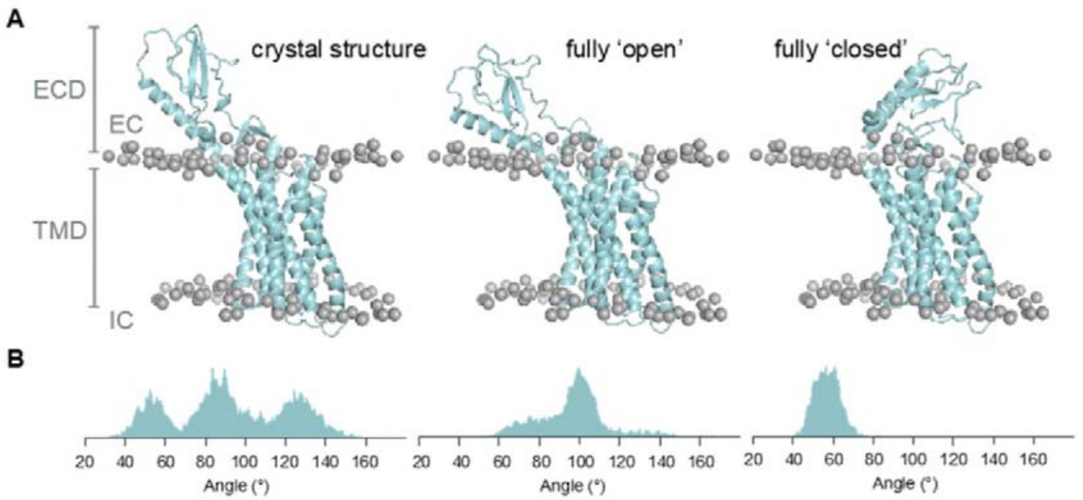

Supplementary Figure S2: Structures used in atomistic simulations. A) Comparison of the initial conformations used in atomistic simulations of $GCGR_{apo}$ (light blue). Each conformation was simulated for 2 x 500 ns in a bilayer composed of POPC (65%): GM3 (10%): CHOL (25%) in the extracellular leaflet and POPC (65%): PIP_2 (10%): CHOL (25%) in the intracellular leaflet. The fully 'open' and fully 'closed' conformations were backmapped from the end of coarse-grain simulations using the *backward.py* and *initram.sh* scripts. Lipid phosphate groups are shown as grey spheres and the position of the extracellular (EC) and intracellular (IC) membranes are marked. **B)** ECD-TMD angle distribution across each of the simulations setups defined as the angle between two planes formed by the Cα beads of R199, V285 and T369 on the TMD, and E34, H45 and H93 on the ECD.

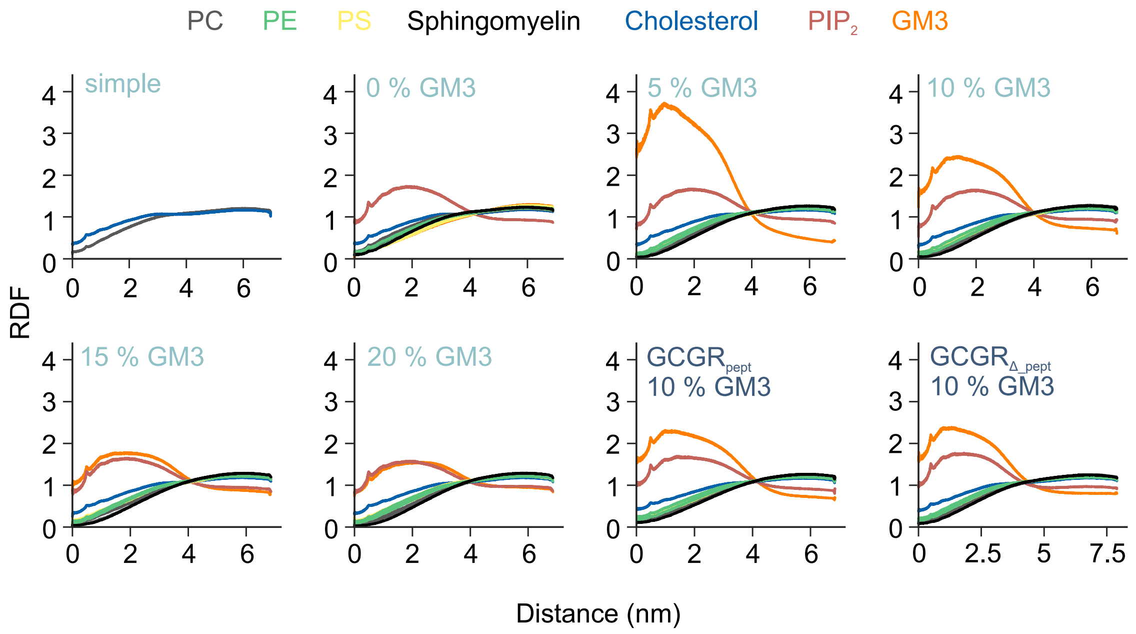

Supplementary Figure S3: Preferential distribution of GM3 and PIP_2 around GCGR. Radial distribution function (RDF) of lipid species surrounding the GCGR TMD centre of geometry in the *xy* plane during CG simulations of $GCGR_{apo}$ (light blue), $GCGR_{pept}$ and $GCGR_{\Delta\_pept}$ (dark blue). $GCGR_{apo}$ was embedded in simple or complex bilayers containing (0-20% GM3). $GCGR_{pept}$ and $GCGR_{\Delta\_pept}$ were embedded in a complex bilayers containing 10% GM3. See Table 1 for a detailed description of lipid compositions and simulation times.

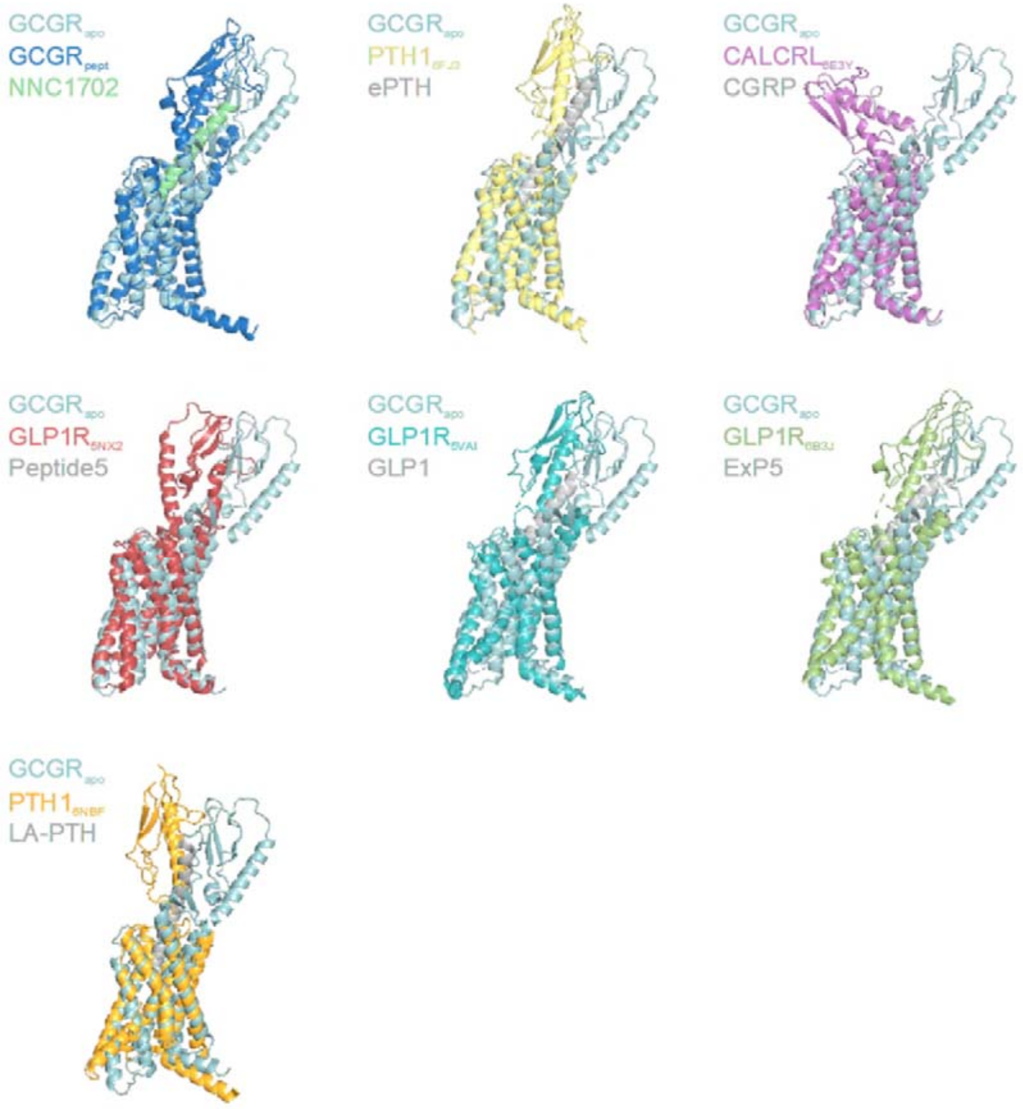

Supplementary Figure S4: Dynamic conformations of Class B1 GPCR ECDs observed in structures. Peptide bound multi-domain Class B1 GPCR structures of the glucagon (GCGR$_{pept}$, PDB: 5YQZ), parathyroid hormone 1 (PTH1, PDBs: 6FJ3, 6NBF), calcitonin receptor-like (CALCRL, PDB: 6E3Y) and glucagon-like receptor 1 (GLP1R, PDBs: 5NX2, 5VA, 6B3J) receptors aligned to the TMD of GCGR$_{apo}$ (PDB: 5XEZ) demonstrating the diversity in ECD position observed experimentally. Peptides bound within the TMD pocket are coloured grey with the exception of NNC1702 which is coloured lime green.

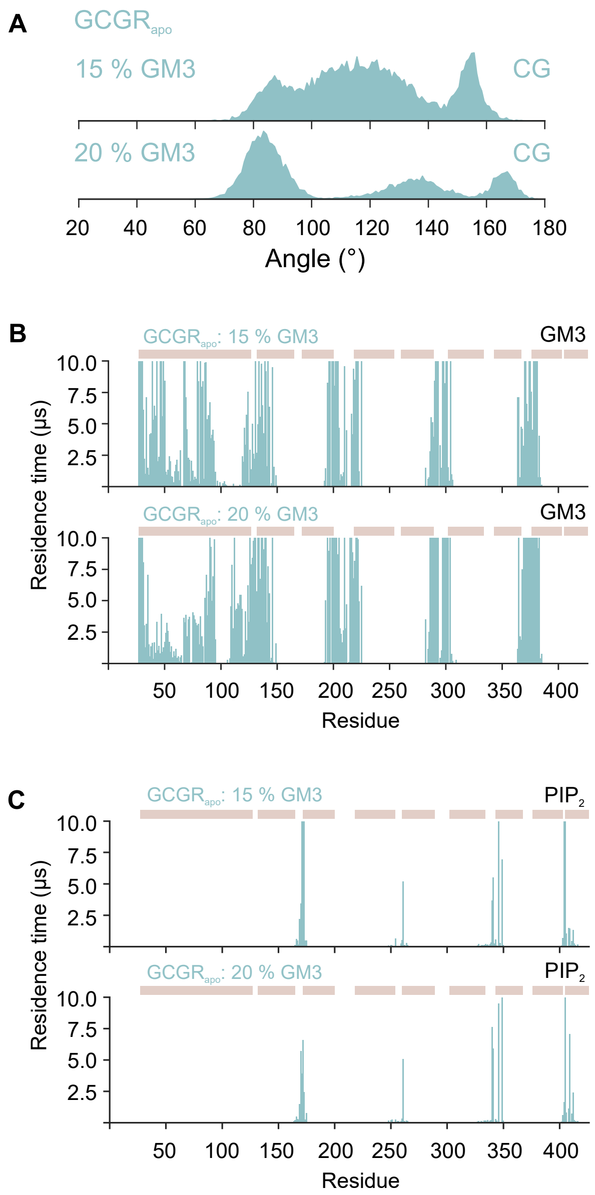

Supplementary Figure S5: ECD dynamics and lipid contacts in bilayers containing 15% or 20% GM3. Analysis derived from CG simulations of $GCGR_{apo}$ embedded in complex bilayers containing 15-20% GM3. **A)** ECD-TMD angle distribution across the simulations defined as the angle between two planes formed by the backbone beads of R199, V285 and T369 on the TMD and E34, H45 and H93 on the ECD. **B)** GM3 and **C)** PIP_2 headgroup interaction profiles with $GCGR_{apo}$ in CG simulations. Lipid headgroup residence times were calculated using an in house procedure (PyLipID) with a 0.55 nm and 1.0 nm dual cut-off scheme. The position of the ECD, TM1-7 and H8 are shown above the contact profile as ochre rectangles.

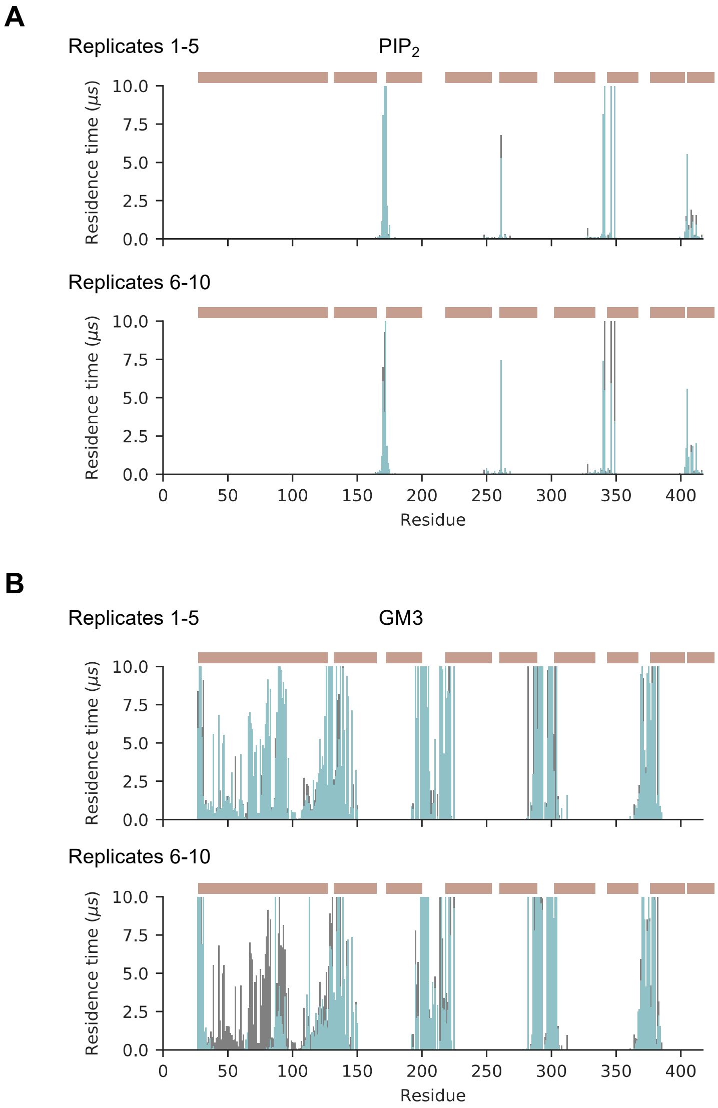

Supplementary Figure S6: Convergence of GM3 and PIP_2 residence times in CG simulations. Lipid interaction residence times of **A)** PIP_2 and **B)** GM3 headgroups from 10 x 10 µs simulations of $GCGR_{apo}$ embedded in a complex bilayer containing 10 % GM3 (grey) overlaid with residence times when simulations were split into two 5 x 10 µs groups (light blue). A 0.55 and 1.0 nm dual interaction cut-off was employed for lipid headgroup contacts analysis. Discrepancies in GM3 headgroup interactions with the ECD in **B** are accounted for by different ECD opening propensities between the two simulation subsets. The position of the ECD, TM1-7 and H8 are shown above the contact profile as ochre rectangles.

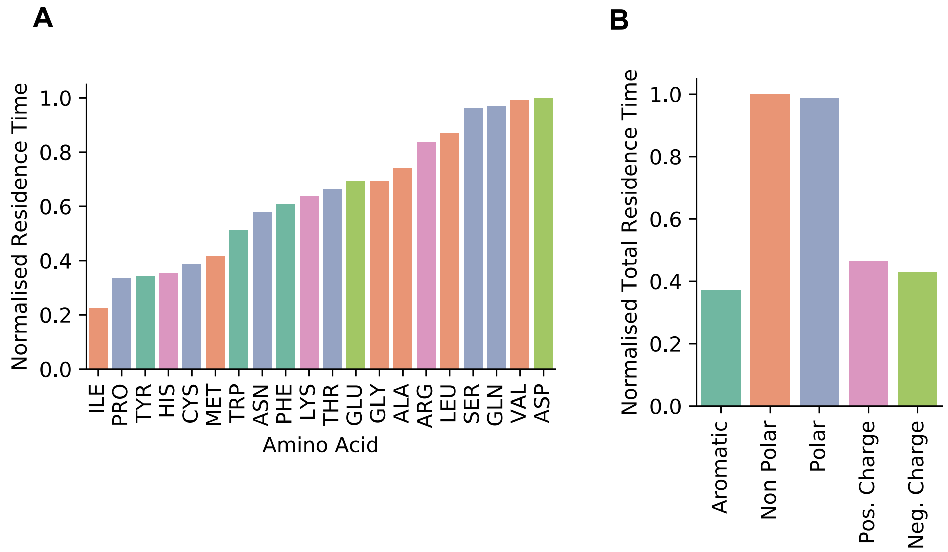

Supplementary Figure S7: Contribution of GCGR amino acid types to GM3 interactions in CG simulations. A) GM3 headgroup residence times for interaction with GCGR amino acids across all CG simulations. Residence times were summed and normalised between 0 and 1 relative to the amino acid with the greatest GM3 headgroup residues time (ASP). Residues are coloured according to physical properties: aromatic (turquoise), non-polar (orange), polar (blue), positively charged (pink) and negatively charged (green). **B)** GM3 residence times grouped according to the physical properties of amino acids. Data for individual amino acids from **A** were grouped according to their physical properties; aromatics (F, Y, W), non-polar (G, A, V, L, M, I), polar (S, T, C, P, N, Q), positively charged (R, K, H) and negatively charged (E, D) and are plotted by total residue type contribution to GM3 interactions. Residence times were normalised between 0 and 1 relative to the amino acid type with the greatest total GM3 headgroup residence times (non-polar).

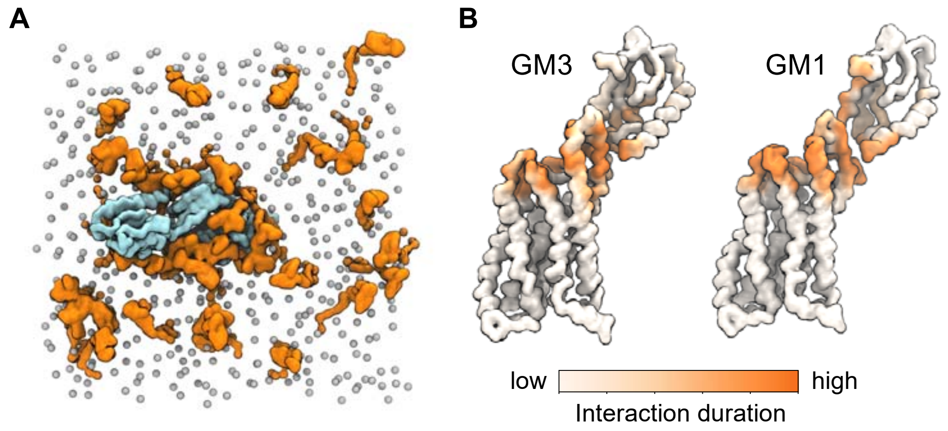

Supplementary Figure S8: Comparison of GM1 and GM3 contacts. CG simulations (3 x 10 µs) of $GCGR_{apo}$ in a 'complex' bilayer were performed whereby 10 % GM1 was used in place of GM3. **A)** Snapshot from the end of one CG simulation showing localisation of GM1 (orange) around $GCGR_{apo}$ (light blue). The membrane patch is shown from above and phosphate groups are shown as grey spheres. **B)** Comparison of GM3 and GM1 contacts mapped onto the structure of $GCGR_{apo}$ showing GM1 interactions over the extracellular TMD regions and the ECD.

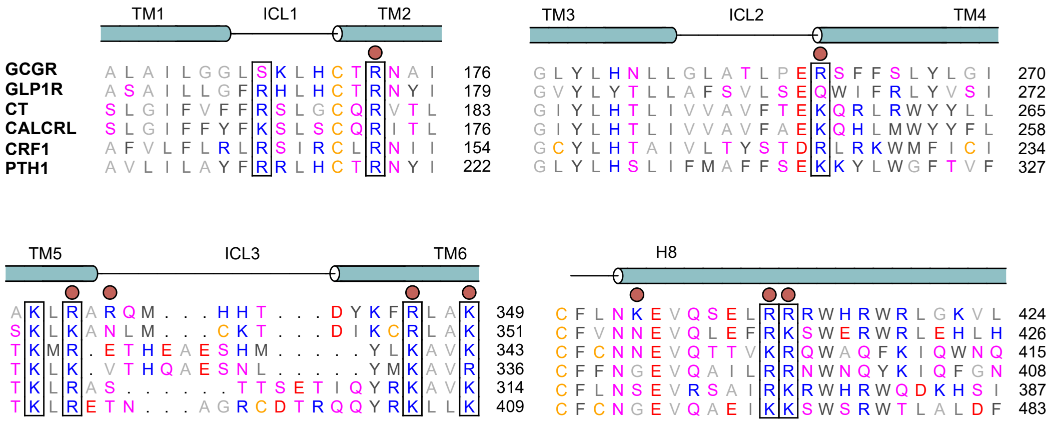

Supplementary Figure S8: Structure-based sequence alignment of the intracellular regions of Class B1 GPCRs. Residues are coloured by residue type and the position of helices in the $GCGR_{apo}$ crystal structure indicated. $GCGR_{apo}$ residues which contribute to PIP_2 binding are marked by red circles. Black rectangles indicate conserved basic residues. Structure based sequence alignment was performed on GPCRdb.org using the human calcitonin (CT), calcitonin receptor-like (CALCRL), corticotropin-releasing factor 1 (CRF1), glucagon-like peptide-1 (GLP1R), glucagon (GCGR) and parathyroid hormone-1 (PTH1) receptors with manual adjustment based on the position of helices observed in structures.
